# UMAMIT14 is an amino acid exporter involved in phloem unloading in Arabidopsis roots

**DOI:** 10.1093/jxb/erw412

**Published:** 2016-11-17

**Authors:** Julien Besnard, Réjane Pratelli, Chengsong Zhao, Unnati Sonawala, Eva Collakova, Guillaume Pilot, Sakiko Okumoto

**Affiliations:** Department of Plant Pathology, Physiology and Weed Science, Virginia Tech, Blacksburg 24061, VA, USA

**Keywords:** Amino acids, Arabidopsis, membrane export, nitrogen, phloem, transporter.

## Abstract

Amino acids are the main form of nitrogen transported between the plant organs. Transport of amino acids across membranes is mediated by specialized proteins: importers, exporters, and facilitators. Unlike amino acid importers, amino acid exporters have not been thoroughly studied, partly due to a lack of high-throughput techniques enabling their isolation. Usually Multiple Acids Move In and out Transporters 14 (UMAMIT14) from Arabidopsis shares sequence similarity to the amino acid facilitator Silique Are Red1 (UMAMIT18), and has been shown to be involved in amino acid transfer to the seeds. We show here that *UMAMIT14* is also expressed in root pericycle and phloem cells and mediates export of a broad range of amino acids in yeast. Loss-of-function of *UMAMIT14* leads to a reduced shoot-to-root and root-to-medium transfer of amino acids originating from the leaves. These fluxes were further reduced in an *umamti14 umamit18* double loss-of-function mutant. This study suggests that UMAMIT14 is involved in phloem unloading of amino acids in roots, and that UMAMIT14 and UMAMIT18 are involved in the radial transport of amino acids in roots, which is essential for maintaining amino acid secretion to the soil.

## Highlight

UMAMIT14, a member of the Usually Multiple Acids Move In and out Transporters 14 family of amino acid transporters, is involved in unloading amino acids from the phloem in roots in addition to a previously described role in seed loading.

## Introduction

In addition to their role as primary metabolites, amino acids are one of the main nitrogenous compounds transported by xylem and phloem between the plant organs (Pate, 1976). In most species, Gln and Asn are the predominant amino acids found in the xylem sap, while all amino acids are transported in the phloem sap (Bollard, 1960; Winter *et al.*, 1992; Lohaus *et al.*, 1994; Lam *et al.*, 1995; Lohaus and Moellers, 2000; Pilot *et al.*, 2004). Amino acid translocation through the plant requires crossing membranes at multiple locations. In roots, xylem loading requires membrane export from the stele cells into the xylem sap. In shoots, apoplasmic phloem loading from the leaf parenchyma requires at least one export and one import step across membranes. By their nature, amino acids are either zwitterions or have a net positive or negative charge, requiring transporters to cross the hydrophobic lipid bilayer at the rates that are observed experimentally (de Jong *et al.*, 1997).

Amino acid transport across membranes is mediated by importers, exporters, and facilitators. Identified plant amino acid importers belong to the families of Amino Acid Permease (AAP), Aromatic and Neutral Transporter (ANT), Cationic Amino acid Transporter (CAT), Lys His Transporter (LHT), Pro Transporter (ProT), and GABA transporter (GAT) (Pratelli and Pilot, 2014). Many of them were isolated by functional complementation of yeast strains lacking endogenous amino acid importers, based on the fact that rescued import function enables growth on media with amino acids as a sole nitrogen source (reviewed in Ortiz-Lopez *et al.*, 2000; Wipf *et al.*, 2002; Rentsch *et al.*, 2007; Pratelli and Pilot, 2014). Several of these importers are involved in long-distance transport of nitrogen. For instance, AtAAP2 is responsible for xylem–phloem amino acid exchange (Zhang *et al.*, 2010), AtAAP8 is involved in seed filling of amino acids during embryogenesis (Schmidt *et al.*, 2007), and AtLHT1 is involved in amino acid uptake by roots and import from the apoplasm into mesophyll cells (Hirner *et al.*, 2006; Svennerstam *et al.*, 2008,^ 2011^). The lack of expression of any of these importers has an impact on plant growth, development, or yield, emphasizing the importance of amino acid importers in nitrogen long-distance transport.

In contrast, in spite of physiological evidence for amino acid export activity, very few exporters have been identified due to a lack of efficient screening techniques (Okumoto and Pilot, 2011). The first report on a molecular mechanism regulating amino acid export came from the study of Arabidopsis *Glutamine Dumper 1* (*AtGDU1*). AtGDU1 is not a transporter, but its over-expression triggers Gln secretion from the hydathodes, suggesting that it functions as a positive regulator of amino acid exporters (Pilot *et al.*, 2004; Pratelli *et al.*, 2010). Arabidopsis Bidirectional Amino Acid Transporter 1 (AtBAT1) has been shown to mediate import of GABA, Arg, and Ala, and export of Lys and Glu at the mitochondrion membrane (Dundar and Bush, 2009; Michaeli *et al.*, 2011). Siliques Are Red1 (SiAR1) has been characterized as a bi-directional amino acid transporter responsible for amino acid accumulation in developing siliques (Ladwig *et al.*, 2012). SiAR1/UMAMIT18 is a member of the Usually Multiple Acids Move In and out Transporters family [UMAMIT, also called *Medicago truncatula* Nodulin 21 (MtN21); Denancé *et al.*, 2014], belonging to the Drug/Metabolite Transporter superfamily. Walls Are Thin1 (WAT1/UMAMIT05) has been shown to be a tonoplastic transporter for auxin, a molecule with a chemical structure similar to Trp (Ranocha *et al.*, 2013). UMAMIT14 has recently been shown to mediate both import and export of amino acids, and to be involved in amino acid loading in seeds (Müller *et al.*, 2015). Here, we report the functional characterization of UMAMIT14 as an amino acid exporter in yeast and its involvement in unloading amino acids from the phloem in roots.

## Material and methods

### Plant culture

Arabidopsis plants (Col-0) were grown in long days (16 h light at 50 μmol m^–2^ s^–1^ at the soil surface, 50% humidity, and 22 °C) or short days (12 h light, 35 μmol m^–2^ s^–1^ at the soil surface, 35% humidity, and 22 °C) in soil composed of a 2:1 ratio of Sunshine Mix™ and Vermiculite. Plants were watered with 0.15 g l^–1^ MiracleGro™ fertilizer (24:8:16, N:P:K) three times a week. Arabidopsis plants were also grown in hydroponic conditions as described previously (Pratelli *et al.*, 2016). For growth *in vitro*, Arabidopsis seeds were sown on ‘J medium’ [2 mM KNO_3_, 1 mM CaSO_4_, 1 mM KH_2_PO_4_, 0.5 mM MgSO_4_, 50 μM NaFeEDTA, 50 μM H_3_BO_3_, 12 μM MnCl_2_, 1 μM CuCl_2_, 1 μM ZnCl_2_, 30 nM (NH_4_)_6_Mo_7_O_24_; Lejay *et al.*, 1999] supplemented with 20 mM KNO_3_ in 0.8% agar with pH adjusted to 5.8 with KOH. For hygromycin selection, J medium was supplemented with 20 μg ml^–1^ hygromycin, and for kanamycin selection it was supplemented with 50 μg ml^–1^ kanamycin and 30 mM sucrose. Plants were transformed using the floral dip method using *Agrobacterium tumefaciens* strain GV3101 (pMP90) (Clough and Bent, 1998). The *umamit14* T-DNA line SALK_037123 (*umamit14-1*) was obtained from the Arabidopsis Biological Resource Center (Alonso *et al.*, 2003), and the T-DNA insertion was confirmed by PCR (see Supplementary Fig. S1A at *JXB* online). To generate the complemented line, *umamit14-1* was transformed with *UMAMIT14* promoter–*UMAMIT14 cDNA-Venus*. Nitrogen starvation experiments were performed as described in Supplementary Fig. S2.

### DNA constructs

The *UMAMIT14* promoter (–1903 bp upstream from ATG) or the *UMAMIT14* promoter and genomic sequence were PCR-amplified from Col-0 genomic DNA. The PCR fragments were cloned into pDONRZeo (Life Technologies, USA). The *UMAMIT14* promoter or *UMAMIT14* genomic sequence were recombined into the destination vectors pWUTkan2 or pPWGYTkan, respectively, derivatives of pJHA212K (Yoo *et al.*, 2005; see Supplementary Fig. S3), generating the plasmids used for *UMAMIT14 promoter–GUS* studies and transient expression of *35S:UMAMIT14–GFP* in Arabidopsis cotyledons. To generate *UMAMIT14* promoter–*UMAMIT14* cDNA-*Venus*, *UMAMIT14* cDNA without the stop codon was amplified by RT-PCR and cloned into a modified pENTR1A vector containing Venus (Price *et al.*, 2013). The *UMAMIT14* cDNA-*Venus* sequence was transferred into a modified pPWYTkan (pJHA212K-derived), in which the *35S* promoter was replaced with the *UMAMIT14* promoter (Supplementary Fig. S3). For yeast uptake studies, *UMAMIT14* cDNA was cloned into the pDONRZeo vector and was transferred to the yeast expression vectors pDR196-Ws (Loque *et al.*, 2007), pAP-Ws, and pAP-Ws-AAP3. For the empty vector controls, the gateway cassettes from pDR196-Ws, pAP-Ws, and pAP-Ws-AAP3 were removed by Gateway cloning with a pDONR vector containing only a stop codon between the attL sites (Supplementary Fig. S4). All entry clones were sequenced prior to use. Sequence information for all vectors is available upon request. Primers used for cloning and qRT-PCR are listed in Supplementary Table S1.

### Analytical methods

Shoots and roots of 5-week-old Arabidopsis plants grown in hydroponic conditions were harvested and frozen in liquid nitrogen, lyophilized, and pulverized. Total free amino acids were extracted by adding 400 μl of chloroform and 10 mM HCl (1:1 mixture) to 0.5–2 mg of plant tissue in a tube containing 0.2 nmol of dry norvaline. The aqueous phase was collected, the organic phase was re-extracted with HCl and chloroform, and the supernatants were pooled. Phloem sap was obtained as described in Corbesier *et al.* (2001) from the leaves of 5-week-old Arabidopsis plants grown in soil in long days. Amino acids were analyzed via Ultra Performance Liquid Chromatography (UPLC; Waters, USA), as described in Collakova *et al.*, (2013). Amino acid content in root and shoot samples was normalized against dry weight and norvaline content. Amino acid content in phloem exudates was normalized against K^+^ content, determined using an Inductively Coupled Plasma Atomic Emission Spectrophotometer (Analytical instruments, USA). Carbon and nitrogen contents of 2 mg of dry seeds were measured using the dry combustion method with a CE Instruments NC 2100 elemental analyzer (ThermoQuest, Italy) at the Duke Environmental Stable Isotope Laboratory, Duke University, NC. Seed protein extraction was performed as described by Gallardo *et al.* (2002) using 1 mg of seeds, and proteins were quantified by the Bradford assay (Bradford, 1976).

### Phloem transfer and seedling secretion assays

For the shoot-to-root transfer assays, 5-week-old Arabidopsis plants grown in hydroponic conditions were removed from the tip boxes, and sink leaves, defined as leaves with a surface area <25% of the largest leaf, were removed (Supplementary Fig. S9A). This largest leaf was then cut around the mid vein and dipped into a 1.5-ml tube containing 1.5 ml of J medium with either 2 mM sucrose + 2 mM Gln + [^3^H]Gln, or 2 mM sucrose + [^14^C]sucrose with a final specific activity of 24.4 kBq µmol^–1^ in the uptake solution for Gln or sucrose. Roots were dipped in an adjacent 1.5-ml tube containing 1.5 ml of J medium. After 4 h, the fed leaf, shoots, roots, and medium bathing the roots were harvested separately. Shoots and roots were dried, weighed and bleached in 500 µl 5% NaClO. Radioactivity in shoots, roots, and root bathing medium was counted using a LS 6500 Multipurpose scintillation counter (Beckman Coulter, USA). To analyze the amino acids secreted from Arabidopsis seedlings, 10 Arabidopsis seeds were germinated in 24 well-plates containing 1 ml per well of J medium supplemented with 20 mM KNO_3_ and 30 mM sucrose, with pH adjusted to 5.8 with KOH. After 2 weeks, the medium was replaced with 1 ml of fresh medium and plants were grown for three more days. The medium was harvested, lyophilized, and resuspended in 300 µl UPLC-grade water, and amino acid content was analyzed by UPLC as described above. Content was normalized using plant dry weight.

### Yeast-based assays

GNP1 (YDR508C) and AGP1 (YCL025C) were sequentially deleted from the genome of 22Δ8AA (Fischer *et al.*, 2002) using the loxP-kanMX-loxP disruption cassette (Guldener *et al.*, 1996) to create 22Δ10α. Uptake of radiolabeled amino acids was performed according to Su *et al.* (2004). For the yeast secretion assay, cells were grown for 22 h in a minimal medium (Jacobs *et al.*, 1980), the OD was recorded, the medium was separated from the cells by filtration, and then clarified on a 10-kDa exclusion membrane. Amino acid content of the medium was determined by UPLC as described above.

### Uptake in planta

Two-week-old Arabidopsis plants were grown on a vertical plate containing half-strength Murashige and Skoog (MS) medium supplemented with 30 mM sucrose in 1% agar. Roots and shoots were separated and each sample was transferred into 1-ml half-strength MS medium supplemented with 30 mM sucrose for 5 h under agitation at 300 rpm. Uptake lasted 10 min in a solution containing 1 ml of half-strength MS medium supplemented with 30 mM sucrose and 2 mM glutamine + [^3^H]Gln with a final specific activity of 37 kBq µmol^–1^. Efflux was performed as described by Pratelli *et al.* (2010) for 20 min. Radioactivity was then measured for each root, shoot, root bathing and shoot bathing media.

### RNA extraction and qRT-PCR

RNAs were extracted using the RNAeasy plant kit (Qiagen, USA) according to the manufacturer’s recommendations. Samples of 2 µg of total RNA were used for cDNA synthesis with random primers using a High Capacity cDNA Reverse Transcription Kit (Applied Biosystems, USA). qRT-PCR was performed using SyBR® Green PCR Master Mix in a 7500 Real Time PCR System (Applied Biosystems, USA) according to manufacturer’s recommendation.

### GUS assay and cross-sections

GUS assays were performed on 2-week-old Arabidopsis seedlings or flowers on 6-week-old plants as described by Martin *et al.* (1992). Stained roots were fixed in 5% glutaraldehyde overnight, followed by dehydration in increasing concentrations of ethanol (30, 50, 60, 70, 80, and 90%, 1 h each). Histochemical GUS analysis was performed by embedding the stained roots into Technovit 7100 resin (Kulzer, Germany) following the manufacturer’s recommendation and slicing the tissues to 1-µm sections using a Leica® Ultracut UCT microtome. The sections were stained with periodic acid (0.5%) and Schiff reagent (5 mM basic fuchsin, 20 mM anhydrous sodium metabisulfite in 0.1 mM HCl).

### Arabidopsis transient expression and confocal microscopy

Arabidopsis transient expression was performed according to Wu *et al.* (2014) using *Agrobacterium* strain C58C1 (pCH32) co-transformed with pPWGYTkan containing the *UMAMIT14* genomic sequence without the stop codon, and a mCherry-expressing vector. After co-incubation, plants were imaged using a Zeiss® LSM 880 confocal laser scanning microscope. Images were taken using wavelengths appropriate for mCherry (543 Ex/600–650 Em), chlorophyll A (405 Ex/650–700 Em), and GFP (488 Ex/490–535 Em). Images obtained were merged using ZEN® software (Zeiss).

### Statistical analyses

One-way ANOVA in conjunction with Tukey’s test or *t*-tests were used to determine significant differences (*P*<0.05) between samples in JMP (SAS, USA).

### Accession numbers

Accession numbers for UMAMIT14 (AT2G39510) and UMAMIT18/SiAR1 (AT1G44800) have been assigned by The Arabidopsis Information Resource (TAIR; http://www.arabidopsis.org/).

## Results

Plant amino acid transporters have typically been characterized by expression in yeast strains, particularly 22Δ8AA, lacking eight plasma membrane amino acid transporters (Fischer *et al.*, 2002). 22Δ8AA is unable to grow on media containing Asp, Glu, Pro, Citrulline, and GABA as the sole nitrogen source at the supplied concentration. The main limitation of this strain is the low number of amino acids on which it can be tested for growth. We improved this strain by deleting two more transporters, Glutamine Permease 1 (GNP1) and Asparagine Glutamine Permease 1 (AGP1), shown to be necessary for transporting Gln and Thr (Velasco *et al.*, 2004; Couturier *et al.*, 2010), yielding strain 22Δ10α. 22Δ10α was unable to grow on any proteinogenic amino acid or GABA except for Arg as the sole nitrogen source (see Supplementary Fig. S5). Because Lys, His, and Cys do not support growth of the parental wild-type strain 23344c and hence 22Δ10α, they are not usable for complementation assays at the tested concentrations. The import of radiolabeled amino acids (Gln, Ala, Pro, and Leu) into 22Δ8AA and 22Δ10α transformed with the empty vector was also compared over time. The background import for Gln and Ala by 22Δ10α was lowered compared to 22Δ8AA, but not for Pro and Leu (Supplementary Fig. S6). This strain therefore provides a better tool for transport assays when compared to 22Δ8AA.

### UMAMIT14 functions as an amino acid exporter in yeast cells

**Fig. 1.**
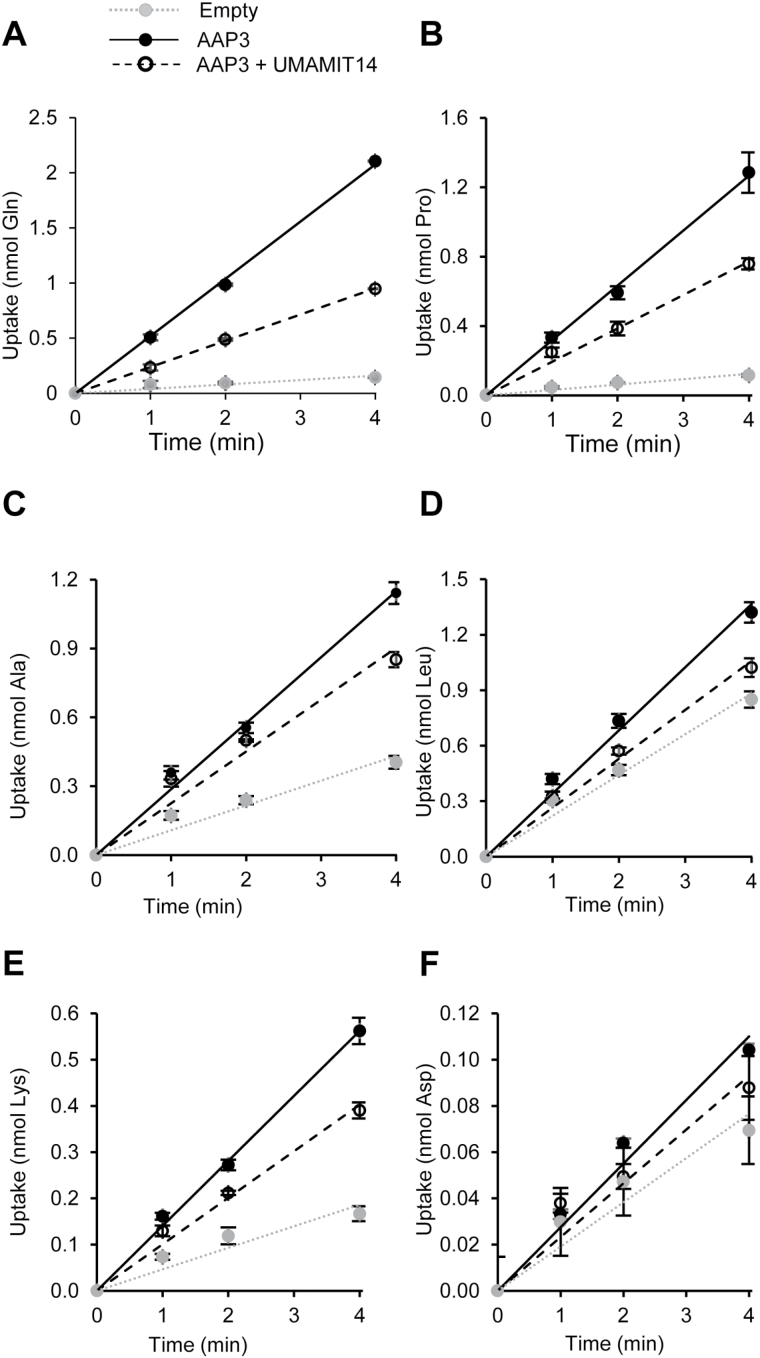
Uptake of radiolabeled amino acids by 22Δ10α yeast cells. Empty: cells transformed with the empty vector pDR196-Ws from which the gateway cassette has been removed. AAP3: cells expressing AAP3. AAP3 + UMAMIT14: cells co-expressing AAP3 and UMAMIT14 carried on a single vector. Uptake of 2 mM of Gln (A), Pro (B), Ala (C), Leu (D), Lys (E), or Asp (F) was examined. Error bars show standard deviation (*n*=3 technical replicates).

The 44 previously cloned Arabidopsis *UMAMIT* cDNAs (Jones *et al.*, 2014) were expressed in 22Δ10α, and the cells were screened for Gln secretion (S. Okumoto, unpublished data). Increased Gln secretion from cells expressing UMAMIT14 suggested that the protein was endowed with an amino acid exporter activity. This result is in good agreement with Müller *et al.* (2015), who reported an increased export of [^14^C]Gln from *Xenopus* oocytes expressing UMAMIT14. We sought to develop a system using 22Δ10α cells that allows facile detection of amino acid export activities. We reasoned that, similar to UMAMIT18 (Ladwig *et al.*, 2012), expressing an amino acid exporter would decrease the accumulation of amino acids taken up by yeast cells. Wild-type cells, harboring all amino acid importers, were used by Ladwig *et al.* (2012), with the caveat that heterologous expression of an exogenous transporter might disturb the expression/activity of endogenous yeast amino acid transporters, several of which are regulated by amino acid levels at the transcriptional and post-transcriptional levels (Stanbrough and Magasanik, 1995; Didion *et al.*, 1996; Springael and Andre, 1998). To circumvent this problem, UMAMIT14 was co-expressed with the plant amino acid importer AAP3 (Fischer *et al.*, 1995), which is unlikely to be regulated by the general amino acid control or the nitrogen catabolite repression systems of yeast. The expression of AAP3 was driven by the Alcohol Dehydrogenase (ADH1) promoter (Ruohonen *et al.*, 1995), which is also unlikely to be regulated by amino acid levels. 22Δ10α cells expressing AAP3 showed a linear import of Gln, Pro, Ala, Leu, Lys, and Asp, which was significantly higher than that of cells transformed with the empty vector (Fig. 1; Fischer *et al.*, 2002). When AAP3 was co-expressed with UMAMIT14, cells accumulated less Gln, Pro, Ala, Leu, and Lys than 22Δ10α cells expressing only AAP3 (Fig. 1A–F). Asp accumulation was not statistically different between these cells. The decreased amino acid accumulation was not due to lower expression of AAP3, as tested by qRT-PCR (data not shown), while an effect on protein content/activity cannot be excluded. Similar results were obtained using 22Δ8AA with Pro (see Supplementary Fig. S7). Expression of UMAMIT14 in 22Δ10α did not increase import of Pro (Supplementary Fig. S7), Gln, Ala, Leu, Lys, and Asp compared to the empty vector (data not shown), nor did it complement yeast growth on any amino acid supplied at 3 mM as the sole nitrogen source (data not shown). These results suggest that UMAMIT14 functions as an exporter in 22Δ10α.

**Fig. 2.**
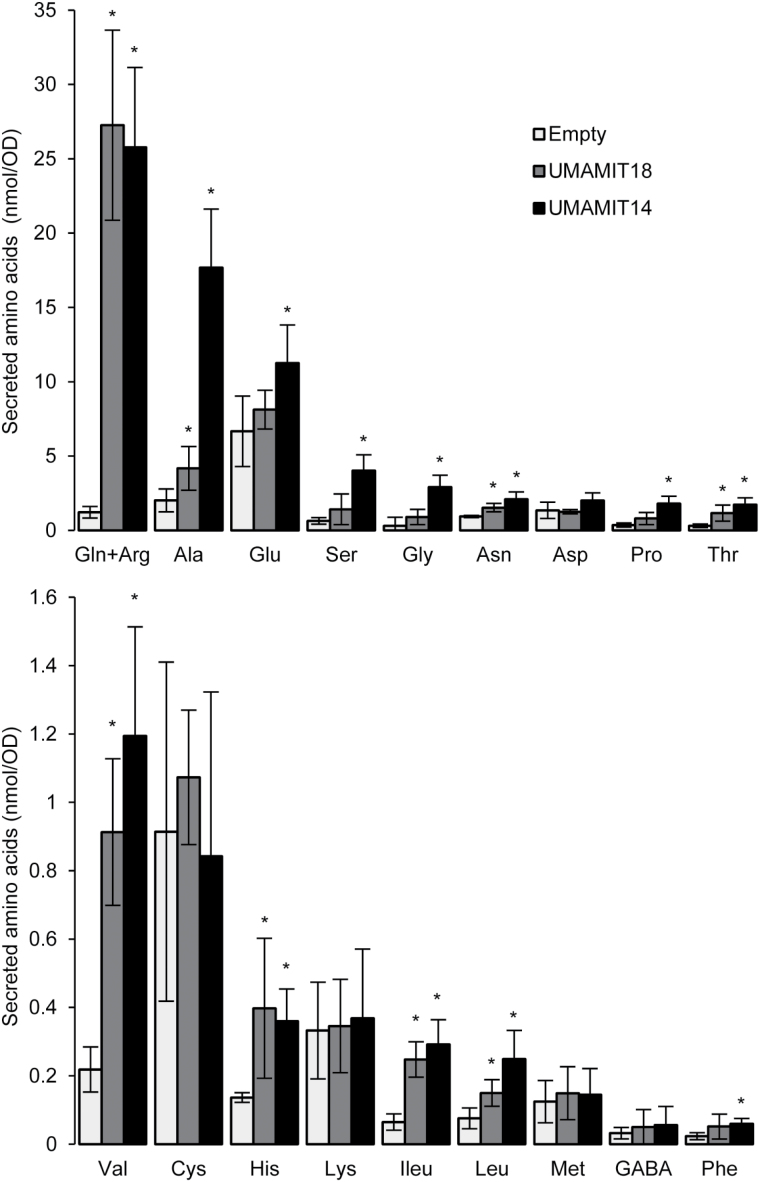
Secretion of amino acids in the medium by 22Δ10α cells. Cells were transformed with the empty vector pDR196-Ws (Empty) from which the gateway cassette had been removed, or containing *UMAMIT14* or *UMAMIT18*. Cells were grown for 22 h in liquid medium and amino acid composition of the medium was determined by UPLC. The Arg peak could not be resolved from the large Gln peak in the *UMAMIT14* and *UMAMIT18* samples so these amino acids are represented by a single bar. For the control, Gln and Arg could be resolved and Gln represents ~50% of the bar height. Error bars correspond to standard deviation (*n*=4 biological replicates). Significant differences compared to the empty vector according to a *t*-test are indicated (*P<0.05).

To confirm this hypothesis, UMAMIT14-expressing cells were grown in the presence of ammonium, and allowed to secrete amino acids into the liquid medium for 22 h, similarly to previous assays (Velasco *et al.*, 2004; Ladwig *et al.*, 2012). The content of Gln+Arg, Ala, Glu, Ser, Gly, Asn, Pro, Thr, Val, His, Ile, Leu, and Phe was increased by expression of UMAMIT14 compared to cells expressing the empty vector (Fig. 2). The results obtained with UMAMIT18 were comparable to those described by Ladwig *et al.* (2012). Therefore, the secretion assays showed that UMAMIT14 behaves as an amino acid exporter in yeast cells under the conditions tested.

Amino acid transport specificity of UMAMIT14 and UMAMIT18 cannot be directly interpreted from these results because the activity/expression of each protein in yeast may be different. To circumvent this problem, the content of secreted amino acids was normalized to the amino acid levels for the empty plasmid control and to the total amount of secreted amino acids (see Supplementary Table S2). Gln, Val, and Ile represented a larger proportion of the secreted amino acids for UMAMIT18 compared to UMAMIT14. Ala, Glu, Ser, Gly, Asp, Pro, and Leu represented a larger part of the secreted amino acids for UMAMIT14 compared to UMAMIT18, suggesting that the amino acid specificity of the two transporters is different.

### UMAMIT14 is a plasma membrane protein expressed in the root phloem and pericycle

**Fig. 3.**
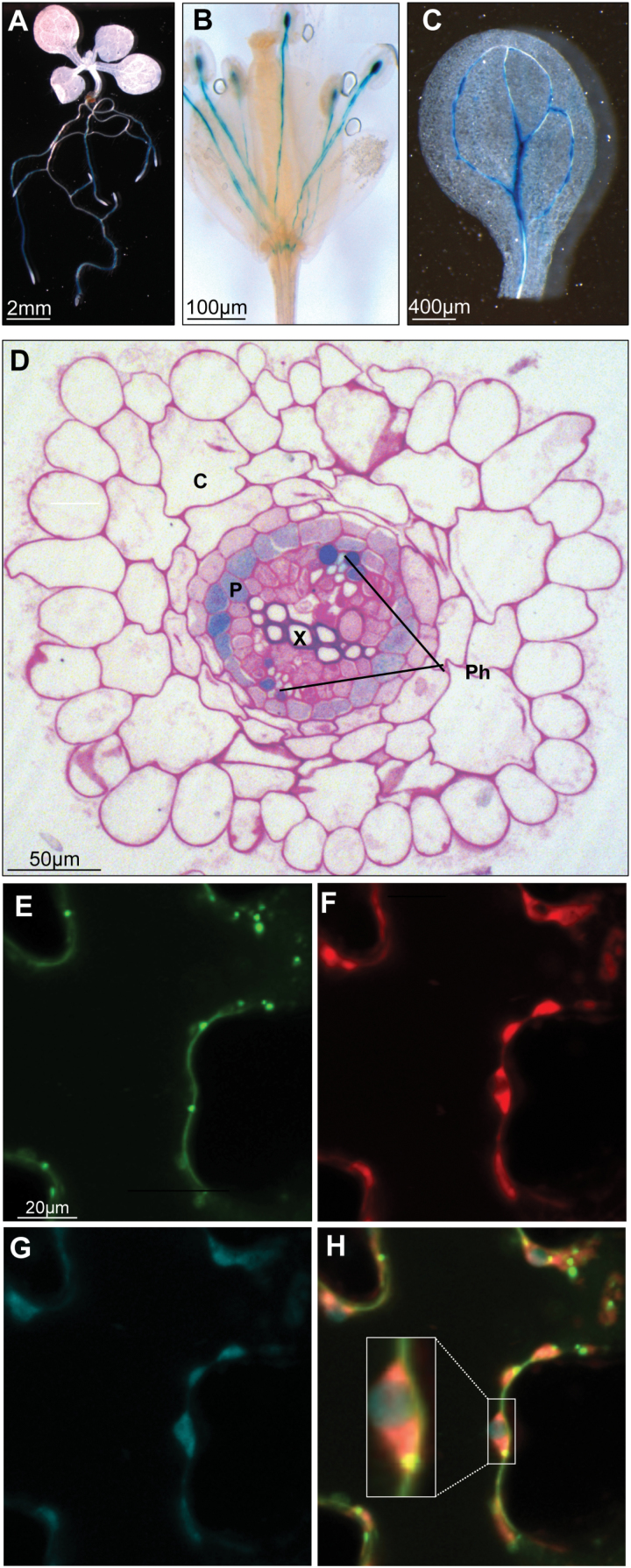
Localization of *UMAMIT14* expression in Arabidopsis. (A–D) Histochemical analysis of GUS activity from Arabidopsis plants expressing *UMAMIT14 promoter–GUS*. GUS staining in the roots of 2-week-old seedling (A), in the flower of a 35-d-old plant (B), and in a 2-week-old cotyledon (C). (D) Cross-section of a 2-week-old root in the maturation zone. Cell walls were stained in pink by the Schiff reagent. C, cortex; Ph, phloem; P, pericycle; X, xylem. Tissues were stained for GUS activity at 37 °C for 16 h for (A) and for 30–45 min for (B–D). (E–H) Subcellular localization of UMAMIT14-GFP in epidermal cells of 2--week-old Arabidopsis cotyledons. (E) UMAMIT14-GFP, (F) mCherry, (G) chlorophyll A, and (H) merged image.

**Fig. 4.**
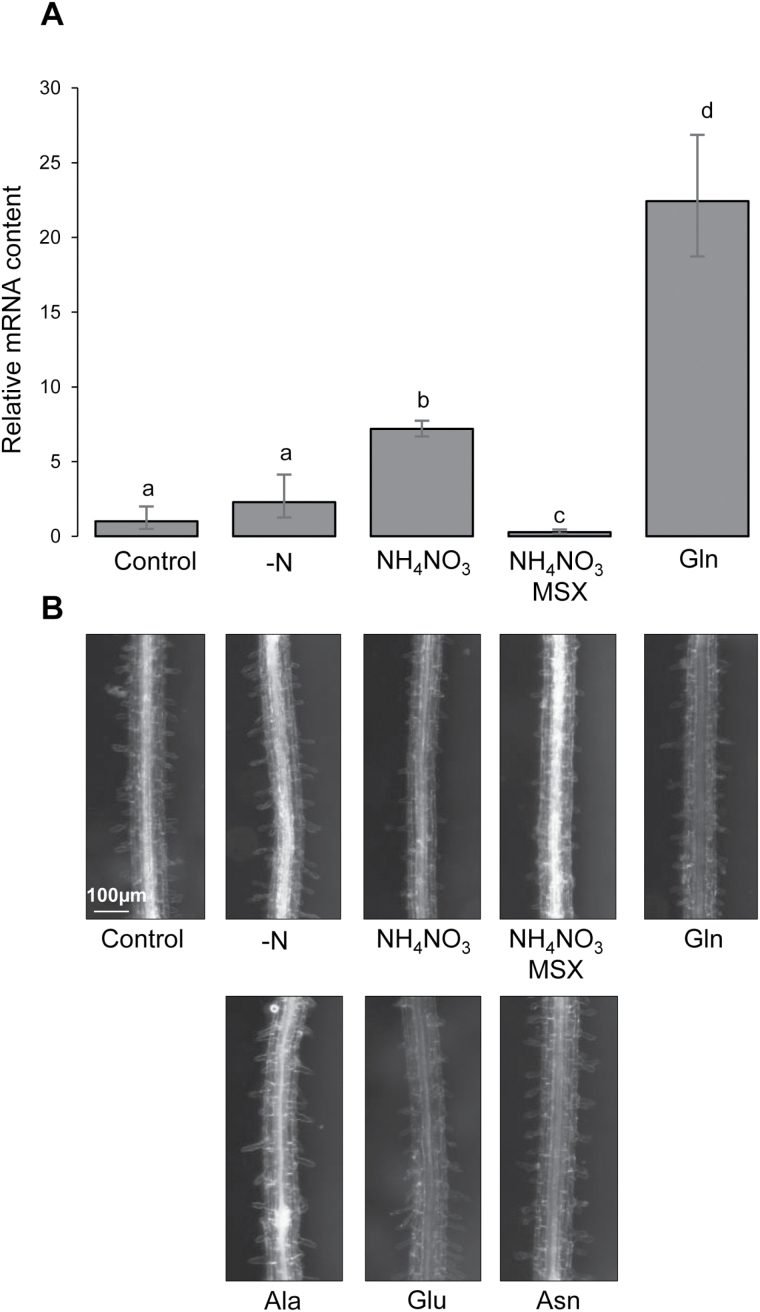
*UMAMIT14* transcript levels in plants grown in different nitrogen conditions. Treatments are described in Supplementary Fig. S10. (A) *UMAMIT14* mRNA levels were determined by qRT-PCR and normalized against *ACTIN2* mRNA levels. Data show the fold-change relative to the non-treated control plants (Control). Error bars correspond to the standard deviation (*n*=3 biological replicates). Significant differences (*P*<0.05) are indicated by different letters according to one-way ANOVA in conjunction with Tukey’s test. (B) GUS activity in the roots of *UMAMIT14 promoter–GUS* plants subjected to the same treatments as in (A), and addition of Ala, Glu, and Asn (20, 20, and 10 mM, respectively). Results are shown from one representative GUS line among four that produced similar results. (This figure is available in colour at *JXB* online.)

The expression pattern of *UMAMIT14* was determined by a promoter-GUS approach in Arabidopsis. *UMAMIT14* promoter activity was detected in the pericycle and phloem cells of roots (Figs 3A, D and 4B) and in the stamen filaments (Fig. 3B). Extending the GUS staining reaction revealed a weak activity of the *UMAMIT14* promoter in the veins of young cotyledons (Fig. 3C), but not in mature leaves. In the wild-type, *UMAMIT14* mRNA accumulated 15 times more in roots than in leaves (Table 1), consistent with the promoter-GUS assay results. Subcellular localization of UMAMIT14 was determined by transiently expressing a *35S:UMAMIT14-GFP* construct in Arabidopsis cotyledon cells. UMAMIT14-GFP was almost exclusively localized at the plasma membrane, with some additional fluorescence in intracellular structures (Fig. 3E–H), in good agreement with Müller *et al.* (2015).

**Table 1.** *UMAMIT14* mRNA accumulation in 5-week-old Arabidopsis plants grown in hydroponic conditions. *UMAMIT14* mRNA levels were determined by qRT-PCR and normalized against *ACTIN2* mRNA accumulation. *UMAMIT14* mRNA levels are expressed relative to the wild-type (WT) root sample. Significant differences (*P*<0.05) are indicated by different letters according to one-way ANOVA in conjunction with Tukey’s test (two biological replicates)

Genotype	Roots	Leaves
WT	1 (a)	0.062 (b)
*umamit14-1*	0.079 (b)	0.041 (b)
*umamit14-1 umamit18-1*	0.057 (b)	0.342 (c)
*umamit14-1:UMAMIT14*	49.3 (d)	0.036 (b)

### UMAMIT14 expression is regulated by organic nitrogen in roots

Publicly available transcriptome data from Arabidopsis suggests that *UMAMIT14* expression is regulated by nitrogen status (Krouk *et al.*, 2010; Patterson *et al.*, 2010; Ruffel *et al.*, 2011). In particular, Patterson *et al.* (2010) showed that *UMAMIT14* mRNA accumulation is increased by nitrate and ammonium treatments. Interestingly, this increase is suppressed by the glutamine synthetase inhibitor methionine sulfoximine (MSX), suggesting that metabolites downstream from glutamine synthetase are responsible for it (Patterson *et al.*, 2010). To further test the effect of the plant nitrogen status on *UMAMIT14* expression, Arabidopsis seedlings were starved of nitrogen for 24 h and then supplied with various inorganic and organic nitrogen sources. After 4 h of incubation *UMAMIT14* transcript levels were seven and 24 times higher in plants incubated with ammonium nitrate and Gln, respectively, than in the control (Fig. 4A). At the same time, *UMAMIT14* transcript levels were 10 times lower in ammonium nitrate plus MSX-treated plants than in the control (Fig. 4A). To test if the increase could result from changes in the expression pattern, *UMAMIT14* promoter activity was studied in the same conditions using four independent *UMAMIT14* promoter-GUS lines. *UMAMIT14* promoter activity correlated well with the abundance of *UMAMIT14* transcripts (Fig. 4B), with no change in localization of the activity in roots: the promoter was always active in vascular tissues, without visible staining in the cortex or epidermis. Application of Glu, Asp, and Ala was also tested, and resulted in a similar increase in promoter activity (Fig. 4B). *UMAMIT14* gene expression was thus increased in the stele by organic nitrogen sources, but not by inorganic ones.

### Umamit14 and umamit18 mutants are specifically affected in transfer of amino acids from the shoots to the roots

To characterize the role of UMAMIT14, the *umamit14-1* loss-of-function line was isolated from the SALK collection (SALK_037123; Alonso *et al.*, 2003; see Supplementary Fig. S1A), which showed over a 10-fold reduction in *UMAMIT14* mRNA accumulation (Table 1). Because UMAMIT14 and UMAMIT18 expression overlaps in the root pericycle and their biochemical functions are similar, *umamit14-1* was crossed with *umamit18-1* (Ladwig *et al.*, 2012) to generate a double homozygous mutant *umamit14-1 umamit18-1*. The *umamit14-1* mutant and the double-mutant grew similarly to the wild-type in soil (Supplementary Fig. S1B and Table S3). Total free amino acid content was similar in roots and shoots for the mutants and the wild-type (Supplementary Fig. S8), while the contents of Ala and Asp were slightly increased in roots of *umamit14-1* and the double-mutant (Supplementary Table S4).

**Fig. 5.**
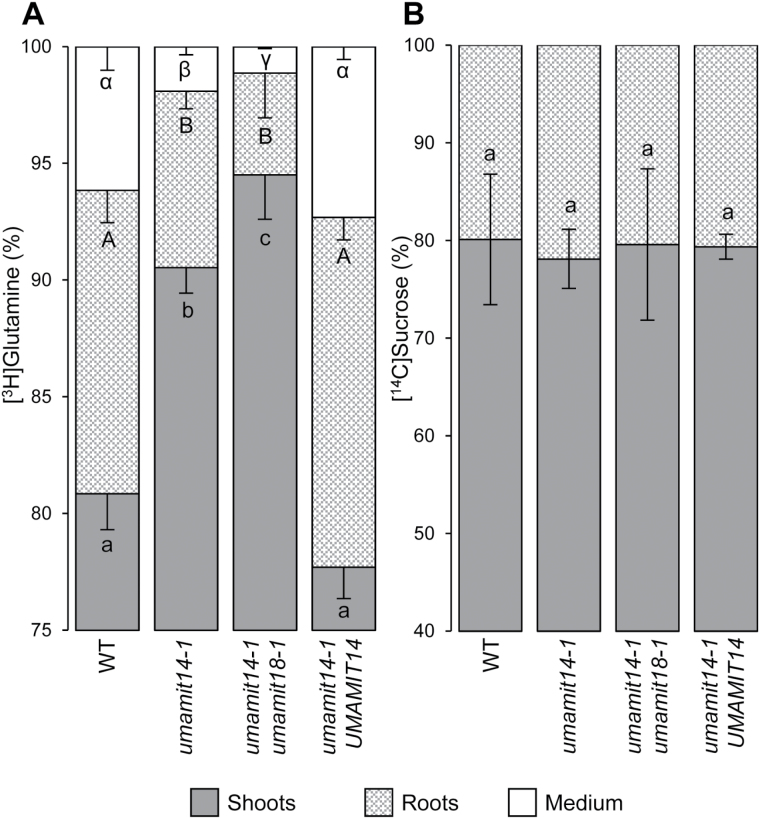
Phloem transfer assay. Distribution of the radioactivity absorbed as [^3^H]Gln (A) and [^14^C] sucrose (B) in shoots, roots, and the growth medium of plants after 4 h of feeding. One mature leaf of 5-week-old plants grown in hydroponics was soaked in J medium containing labeled compounds. The fed leaf was removed from the shoots prior to counting. WT, wild-type. Significant differences (*P*<0.05) are indicated by different letters according to one-way ANOVA in conjunction with Tukey’s test (*n*=3 biological replicates).

Disruption of *UMAMIT14* and *UMAMIT18* is expected to affect amino acid transport. While it might not change the amino acid content at the organ level, it could change amino acid translocation by the long-distance transport systems. Because the *UMAMIT14* promoter was active in the root phloem and pericycle cells, we hypothesized that UMAMIT14 is involved in unloading amino acids from the phloem in roots. A shoot-to-root translocation assay was designed, in which a leaf was fed radiolabeled Gln, and the transfer of the radioactivity to the rest of the rosette, roots, and medium was measured after 4 h (see Supplementary Fig. S9A). Radiolabel translocation from the fed leaf to the roots and from the roots to the medium was reduced by approximately half in *umamit14-1* compared to the wild-type. This decrease was stronger in the *umamit14-1 umamit18-1* double-mutant than in *umamit14-1*, to approximately a third of wild-type level (Fig. 5A). The same trend was observed for the radioactivity secreted into the medium: secretion was reduced from ~6% in the wild-type to ~2% and ~1% in the single- and double-mutants, respectively. Total Gln absorbed by the fed leaf was similar between the mutants and the wild-type, showing that the decreased transfer to the roots is not due to less Gln absorbed by the mutants (Supplementary Fig. S9B). To confirm that the observed decrease was due to the disruption of *UMAMIT14*, the *umamit14-1* mutant was complemented with the *UMAMIT14* cDNA fused to the fluorescent protein Venus (Nagai *et al.*, 2002) under the control of its native promoter, which restored *UMAMIT14* expression as confirmed by qRT-PCR (Table 1). Gln translocation in the shoot-to-root assay in the complemented line was identical to the wild-type (Fig. 5A).

To test whether decreased amino acid translocation to the roots resulted from a decrease in phloem translocation rate and/or in amino acid concentration in the phloem, leaf phloem exudates from the *umamit14-1* and *umamit18-1* single- and double-mutants were collected and analyzed. No difference in amino acid composition was observed between wild-type plants and the three mutants (Table 2). Phloem exudation rate was estimated by measuring K^+^ release from the leaves during this experiment, and no difference was found between the genotypes (Table 2). To further confirm these results, the shoot-to-root translocation assay was performed using radiolabeled sucrose as a proxy for phloem transfer rate. Total absorption by the fed leaf and translocation of the radioactivity to the roots was identical between the wild-type and the *umamit* mutant lines (see Supplementary Fig. S9C and Fig. 5B), indicating that phloem translocation is not affected by the mutations. These results suggest that the *umamit* mutations specifically affect translocation of amino acids from the shoots to the roots, and not phloem sap flow rate.

**Table 2.** Amino acid composition and K^+^ content of phloem exudates of 5-week-old Arabidopsis plants grown in soil in long day conditions. No significant differences were found according to one-way ANOVA in conjunction with Tukey’s test (*P*<0.05, three biological replicates)

	Amino acids (nmol mg^–1^ K^+^)																K^+^ (nmol mg^–1^ DW)
	Ala	Asn	Asp	GABA	Gln	Glu	Gly	His	Ile	Leu	Phe	Pro	Ser	Thr	Val	Total	
WT	18.0	11.6	46.4	46.3	28.4	34.2	4.3	2.4	3.9	5.2	3.2	3.8	19.5	14.7	6.3	248.3	0.43
*umamit14-1*	17.0	15.3	53.0	31.3	40.2	54.1	4.9	3.0	5.6	6.9	4.1	4.0	20.8	16.3	7.9	284.5	0.33
*umamit18-1*	20.4	13.2	47.8	57.4	33.2	37.3	6.6	3.5	4.0	5.7	3.4	5.6	23.0	18.3	6.9	286.4	0.46
*umamit14-1/umamit18-1*	22.4	11.4	42.7	44.3	29.0	34.2	7.6	3.1	3.9	5.3	3.3	5.5	25.3	17.4	6.5	262.0	0.39

Decrease of amino acid supply to the roots could possibly decrease root growth under low nitrogen availability. To test this hypothesis, wild-type and *umamit14-1* plants were grown under high and low nitrate regimes. No sugar was added to the medium to limit nitrate assimilation in the roots (Reda, 2015). Under such conditions, root growth was expected to largely depend on the amino acids supplied from the shoots. And indeed, wild-type plants showed a slower root growth in low concentrations of nitrate than in high nitrate; however, for a given nitrate regime the root lengths of the wild-type and the mutant were similar (see Supplementary Fig. S10), showing that in these conditions loss-of-function of *UMAMIT14* did not affect root growth.

### Secretion of amino acids in the medium is affected in the *umamit14-1 umamit18-1* single- and double-mutants

**Fig. 6.**
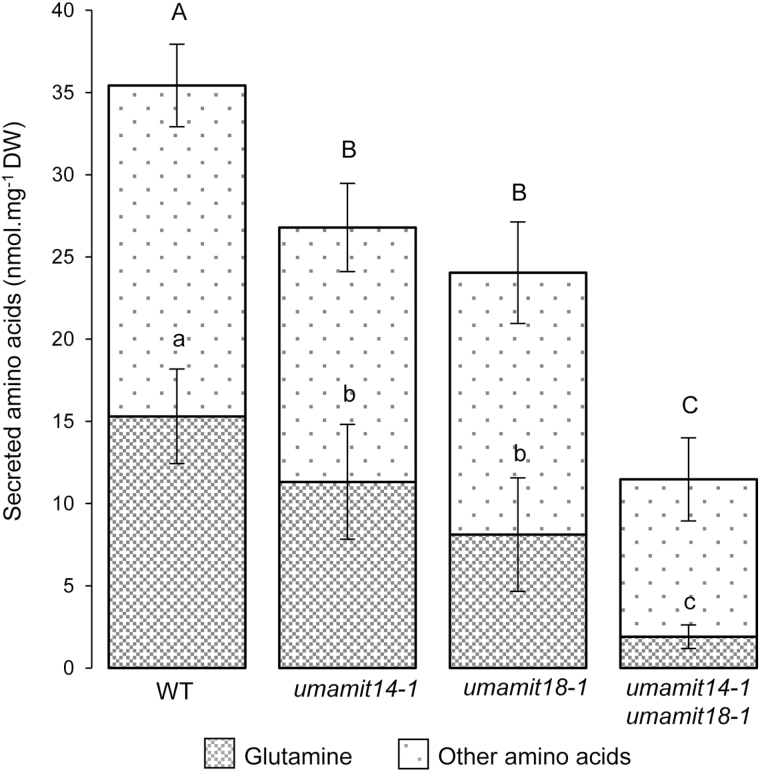
Glutamine and other amino acids secreted by the wild-type (WT), *umamit14* and *umamit18* mutants. Plants were grown for 2 weeks in liquid J medium supplemented with 20 mM KNO_3_ and 30 mM sucrose. The medium was then replaced with fresh J medium and collected after 3 d for analysis. Significant differences (*P*<0.05) are indicated by different letters according to one-way ANOVA in conjunction with Tukey’s test (*n*=6 biological replicates). Contents for individual amino acids from the same dataset are presented in Supplementary Table S5. Amino acid contents were normalized against the dry weight of seedlings in each sample.

**Fig. 7.**
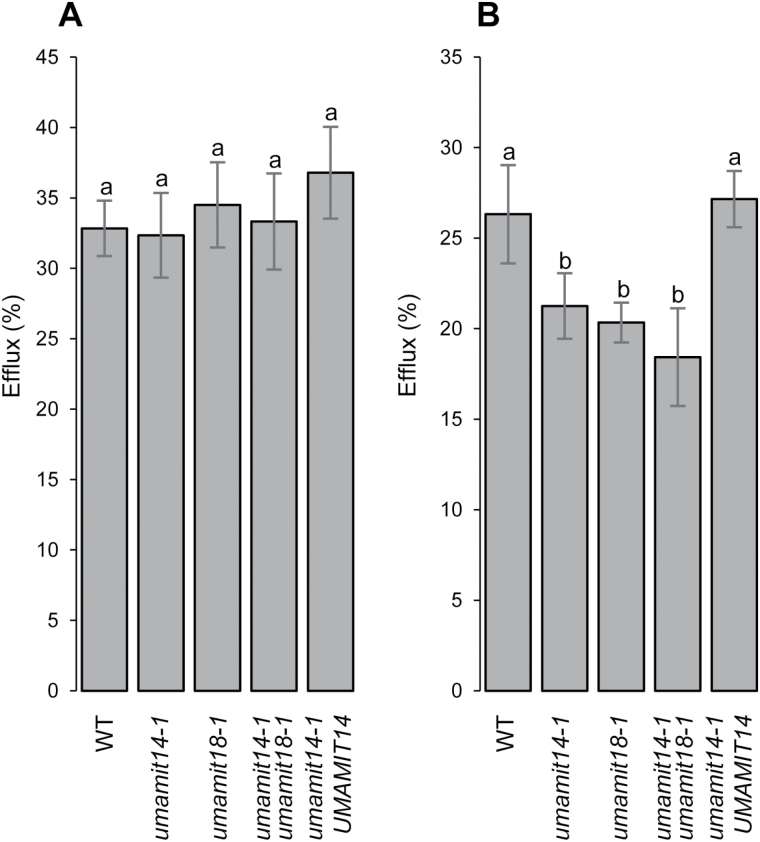
Efflux of [^3^H]Gln by roots and shoots of 2-week-old Arabidopsis seedlings. Uptake and efflux were performed for 20 and 10 min, respectively, using 2 mM Gln. Efflux from shoots (A) and roots (B) are expressed as a percentage of total uptake. Error bars correspond to the standard deviation (*n*=4 biological replicates). Significant differences (*P*<0.05) are indicated by different letters according to one-way ANOVA in conjunction with Tukey’s test.

The shoot-to-root transfer assay showed that less radioactivity is secreted by mutant roots into the medium compared to the wild-type. Since plants secrete a variety of organic molecules into their environment in addition to amino acids (Chaparro *et al.*, 2013), we sought to test whether this label was borne by amino acids or by other compounds, which could be produced from the absorbed [^3^H]Gln. Whole seedlings were grown in liquid medium for 3 d and the amino acid composition of the medium was analyzed. *umamit14-1, umamit18-1*, and *umamit14-1 umamit18-1* secreted, respectively, 25%, 33%, and 72% less amino acids in the medium than the wild-type (Fig. 6 and see Supplementary Table S5). Gln was the most abundant amino acid secreted by the wild-type (47%) and *umamit14-1* (46%), but its abundance was lower for *umamit18-1* (38% of the wild-type) and for the *umamit14-1 umamit18-1* double-mutant (21% of the wild type) (Table S5). This result suggests that most of the radioactive compounds secreted in the medium in the shoot-to-root translocation assay corresponded to amino acids. To specifically test if amino acid secretion by roots is affected by the loss of function of *UMAMIT14* and *UMAMIT18*, both of which are expressed in the root stele, [^3^H]Gln efflux from shoots and roots were measured independently. [^3^H]Gln effluxes from *umamit14-1, umamit18-1*, and *umamit14-1 umamit18-1* roots were decreased compared to the wild-type, while [^3^H]Gln efflux from the shoots remained similar (Fig. 7).

## Discussion

### UMAMIT14 mediates amino acid export in yeast

Two complementary yeast assays were performed to confirm and complete the biochemical properties of UMAMIT14 described by Müller *et al*. (2015). Export activity was first measured in the yeast strain 22Δ10α where UMAMIT14 was co-expressed with AAP3, a well-characterized amino acid importer. Amino acid uptake was lower in cells co-expressing AAP3 and UMAMIT14, compared to the cells expressing AAP3 alone (Fig. 1). In the secretion assay, UMAMIT14-expressing yeast cells were allowed to secrete amino acids into the medium (Fig. 2). 22∆10α cells expressing UMAMIT14 secreted more than four times as much amino acids than control cells. These two assays, enabling measurement of export activity over different time periods, demonstrated that UMAMIT14 displays an amino acid export activity, in good accordance with the results from Müller *et al*. (2015), and reminiscent of UMAMIT18 (Ladwig *et al.*, 2012), UMAMIT11, UMAMIT28, and UMAMIT29 (Müller *et al.*, 2015). Contrary to the report of Müller *et al*. (2015), no import was detected in yeast cells expressing UMAMIT14 when tested by complementation or uptake assays (see Supplementary Fig. S7, and data not shown). It should be noted that Müller *et al.* (2015) found a *K*_m_ value of ~15 mM for import by UMAMIT14 and that high substrate concentrations (up to 100 mM) were used in their assays, compared to 2 mM in our study, probably explaining the different results. Whether UMAMIT14 displays a similar *K*_m_ for export of cytosolic amino acids remains to be determined.

Assuming that cytosolic amino acid content is similar in yeast cells expressing UMAMIT14 or UMAMIT18, comparing the compositions of amino acids secreted in the medium suggests that UMAMIT14 and UMAMIT18 do not have the same amino acid specificities. UMAMIT14 promotes secretion of a wider spectrum of amino acids compared to UMAMIT18 (see Supplementary Table S2). The range of amino acids secreted and the activity to reduce the AAP3-mediated amino acid accumulation (Gln, Pro, Ala, Leu, and Lys; Fig. 1) suggest that, similar to UMAMIT18, UMAMIT14 is a broad-specificity amino acid exporter.

### Possible function of UMAMIT14 in phloem unloading in roots

Publicly available transcriptomic data (Mustroph *et al.*, 2009), promoter-GUS and qRT-PCR results (Fig. 3, Table 1) indicate that UMAMIT14 is expressed in roots in addition to seeds. Subcellular localization and histochemical analysis studies showed that UMAMIT14 is mainly expressed at the plasma membrane and in root pericycle and phloem cells (Fig. 3) in addition to being expressed in seeds (Müller *et al.*, 2015). Because pericycle cells are involved in both xylem and phloem functions (Parizot *et al.*, 2012), we first hypothesized that UMAMIT14 was involved in the transport steps leading to root-to-shoot translocation of amino acids. Root-to-shoot translocation of amino acids first involves export from xylem parenchyma and pericycle cells for loading the xylem. Roles for pericycle-expressed transporters in xylem loading have been previously reported for multiple solutes such as potassium, boron, and auxin (Gaymard *et al.*, 1998; Takano *et al.*, 2002; Kamimoto *et al.*, 2012). If UMAMIT14 or UMAMIT18 were involved in loading amino acids in the xylem, a decrease in xylem amino acid content or transfer rate would have been expected in the *umamit* knock-out lines. The amino acid content of the xylem sap in *umamit14-1* and *umamit14-1 umamit18-1* was not significantly different compared to the wild-type (see Supplementary Fig. S11), nor was the translocation of root-fed amino acids to the shoots (Pratelli *et al.*, 2016). While not supporting an essential role for UMAMIT14 and UMAMIT18 in this process, these results do not exclude their involvement alongside additional transporters. Indeed, publicly available microarray data show that other members of the UMAMIT family (e.g. UMAMIT10, 11, and 17) are highly expressed in the root pericycle (Long *et al.*, 2010).

**Fig. 8.**
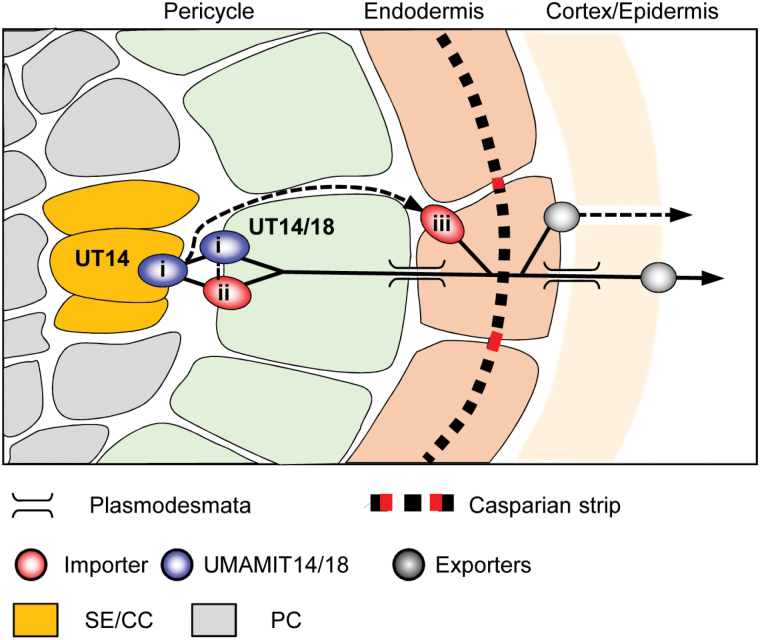
Model of radial transport of amino acids in the mature root. Amino acids coming from the shoots are exported from the sieve elements and companion cells (SE/CC) of the phloem to the stele apoplasm by an exporter (i), possibly UMAMIT14. In the apoplasm, amino acids are taken up by pericycle cells by an importer or by the import ability of UMAMIT14 and UMAMIT18 (ii). Alternatively, amino acids can be taken up from the stele apoplasm by endodermis cells, (iii), by-passing the pericycle uptake. Amino acids can be further exported to the rhizosphere from the endodermis, cortex and/or epidermis cells by presently uncharacterized exporters. Symplasmic and apoplasmic routes are indicated by solid and dotted lines, respectively. PC, parenchyma cells.

Unloading shoot-derived amino acids from the symplasmically isolated phloem into the root stele (Oparka, 1990; Imlau *et al.*, 1999) requires amino acid exporter activity (Fig. 8). Amino acids can then be taken up from the apoplasm by importers into stele cells or be transferred to the xylem towards the shoots (Jeschke and Hartung, 2000). Functional properties and expression of UMAMIT14 in the root phloem cells points towards a role in phloem unloading in roots, a hypothesis supported by our results, which show that less amino acids coming from the shoots are unloaded in root tissues. The analysis of UMAMIT14 expression in seeds led to the hypothesis for a role in unloading amino acids from the phloem to the surrounding tissues (Müller *et al.*, 2015). UMAMIT14 thus appears to be an important phloem unloader, both in roots and seeds. Despite a decrease in phloem amino acid unloading in *umamit14-1* roots, root growth was not significantly affected under the conditions tested (see Supplementary Fig. S11). Nevertheless, the function of UMAMIT14 might become more prominent in other growth conditions. It is noteworthy that organic nitrogen sources applied after a nitrogen starvation period induce the expression of UMAMIT14 (Fig. 4). Nitrogen resupply after starvation has been shown to increase leaf amino acid content within 3 h (Balazadeh *et al.*, 2014). Shortly after amino acid levels increase in shoots, amino acids are probably translocated to the roots to support growth and cell division there. In such a scenario, an increase in the expression of UMAMIT14 would enable higher amino acid unloading into the root tissues. Whether the *umamit14* mutant responds differently from the wild-type under such specific growth conditions remains to be determined.

### Altered shoot-to-root translocation of amino acids decreases the secretion capacity of Arabidopsis roots

Amino acid export from roots measured by amino acid secretion (Fig. 6) and labeled amino acid efflux (Fig. 7) was decreased in the *umamit14* and *umamit18* mutants. Based on these results, we suggest the following model that explains the *umamit14 and umamit18* phenotypes observed in roots (Fig. 8). (1) Altered phloem unloading of amino acids in the *umamit14* mutant increases the local concentration of amino acids in the root phloem; (2) decreased amino acid transport at the pericycle in the *umamit14* and *umamit18* mutants slows down the radial transfer of amino acids from the stele to the peripheral cell layers; and (3) decreased amino acid transfer to the peripheral cell layers in the *umamit14* and *umamit18* mutants decreases the secretion mediated by other amino acid exporters located in the cortex and epidermis. Interestingly, several *UMAMIT* genes are expressed in the cells that are in direct contact with the rhizosphere; for example, UMAMIT04 is expressed in root hairs and atrichoblasts, and UMAMIT06, 37, and 42 are expressed in trichoblasts (Brady *et al.*, 2007). These transporters could mediate the final steps of amino acid secretion out from the root. It should be noted that in the shoot-to-root transfer experiment using [^3^H]Gln, the ratio between Gln retained in the root and Gln secreted into the medium remained similar between the wild-type and the mutants (Fig. 5A). This result suggests that the amino acid efflux capacity from the peripheral cell layers is not affected by the *umamit14* and *umamit18* mutations.

It is well documented that roots secrete various compounds including amino acids into the rhizosphere, which in turn affects the composition of the microbial community (Huang *et al.*, 2014). While root secretion causes net losses of assimilated carbon and nitrogen, root-secreted organic molecules serve important functions such as attracting growth-promoting microorganisms, increasing accessibility to mineral nutrients, and regulating defense against pathogens (Dakora and Phillips, 2002; Huang *et al.*, 2014). For example, it has been shown that amino acids are chemotactic attractants for a wide range of soil-borne bacteria (Simons *et al.*, 1997; Oku *et al.*, 2012; Hida *et al.*, 2015; Webb *et al.*, 2016) and a recent study further suggested that Trp secreted from roots positively regulates auxin production from growth-promoting bacteria, (Liu *et al.*, 2016), which in turn promotes plant growth.

Little is known about where amino acid secretion happens along the length of the root. However, a study using a Trp-sensing bacterial strain showed that Trp secretion from the roots of *Avena barbata* is greatest at 12–16 cm from the root tip, in contrast to sucrose which is secreted mostly from the root tip (Jaeger *et al.*, 1999). In Arabidopsis, secretion of amino acids from roots peaked at a much later stage (28–31 d) compared to that of sugars (7–10 d), also consistent with the secretion from well-developed root sections (Chaparro *et al.*, 2013). Secretion of shoot-derived compounds from a mature section of roots would involve unloading from the phloem, importing into the stele and pericycle cells, radial transfer to the endodermis and cortex through the symplasmic route, and export into the rhizosphere (Fig. 8). Results from the shoot-to-root translocation assay suggested that UMAMIT14 and UMAMIT18 are involved in the translocation of phloem-derived amino acids within roots and ultimately their secretion into the rhizosphere (Fig. 8). Whether the altered amino acid secretion profiles from *umamit* lines impacts the root microbiome remains to be determined.

The role of the UMAMIT14 and UMAMIT18 in the pericycle in relation to the unloading of amino acids to the root tissues is not clear. The pericycle layer is highly connected to the endodermis via plasmodesmata (Ma and Peterson, 2001), suggesting that most solutes will be transferred between these cell layers via the symplasm. Moreover, pericycle cells have been described to be important for transfer of solutes towards the xylem sap via membrane export (see above) and, to our knowledge, not for uptake from the stele parenchyma. It is nevertheless possible that UMAMIT14 and UMAMIT18, for which import activity has been detected in specific conditions (Ladwig *et al.*, 2012; Müller *et al.*, 2015), are responsible for some of the import from the stele apoplasm to the pericycle. The concentration of amino acids in the xylem sap is about 5 mM (see Supplementary Fig. S11; Pilot *et al.*, 2004; Zhang *et al.*, 2010), which may be similar to the concentration of amino acids in the stele apoplasm. At this concentration level, both UMAMIT14 and UMAMIT18 are able to import amino acids into the cytosol, and could then be involved in the radial transfer of amino acids to the root periphery. Additional studies would be needed to test this hypothesis, and to determine the exact roles of UMAMIT14 and UMAMIT18 in pericycle cells.

### Roles of UMAMIT14 and UMAMIT18 in long-distance amino acid transport

In the *umamit14-1* single-mutant, translocation from shoots to roots and root amino acid secretion were reduced compared to the wild-type. Considering the localization of UMAMIT14 in the root phloem and pericycle, the simplest hypothesis to explain the decreased secretion from the roots to the medium in the *umamit14* mutants is that amino acids accumulate in the root phloem and stele due to the decreased export from the phloem to the peripheral layers. Although such a change might increase the amino acid content of the phloem and the stele, it is unlikely that it will affect the overall amino acid content in roots: the decrease in amino acid secretion in *umamit14-1* (estimated at 2.9 nmol mg^–1^ DW d^-1^, based on the data shown in Fig. 6 if the secretion rate from roots is similar to that from whole seedlings) is small compared to the total root amino acid content (393.8 nmol mg^–1^ DW). An increase in the root phloem amino acid concentration might trigger a change in flux so that a larger portion of the amino acids delivered from the leaf phloem are redirected to the xylem before they reach the root, changing the partitioning of amino acids derived from the source leaves (Fig 5A). It is noteworthy that stem/node regions (which were excluded from phloem sap collection) are highly vascularized and are the site for extensive nutrient exchange between the phloem and xylem (Jeschke *et al.*, 1987). We hypothesize that in *umamit14-1* more amino acids are retained within the root phloem and/or the stele due to the lack of exporter activities, and that the resulting increase in root phloem amino acid level triggers a change in amino acid partitioning, potentially within the stem/node region.

In almost all cases, the *umamit14-1 umamit18-1* double-mutant showed an enhanced phenotype compared to the *umamit14-1* mutant: less amino acid transferred to the roots and less amino acid was secreted by the roots. In good agreement with their overlapping expression in the root pericycle and their comparable, albeit distinct, functional properties, UMAMIT18 and UMAMIT14 appear to have similar roles in roots. Unlike UMAMIT14, UMAMIT18 is also expressed in leaf veins (Ladwig *et al.*, 2012), suggesting that it has other roles in the transport of amino acids in the plant. Nevertheless, this role does not seem to affect the composition or velocity of the leaf phloem (Table 2), suggesting that the enhanced effect of the *umamit14 umamit18* double-mutation in shoot-to-root translocation is solely due to the role of UMAMIT18 in the root pericycle.

## Supplementary data

Supplementary data are available at *JXB* online.

Fig. S1. *umamit14-1* and *umamit18-1* T-DNA insertion mutants.

Fig. S2. Nitrogen starvation and recovery experiment set-up.

Fig. S3. Maps of plant vectors used.

Fig. S4. Maps of yeast vectors used.

Fig. S5. Yeast growth complementation assay.

Fig. S6. Amino acid uptake by 22Δ8AA and 22Δ10α.

Fig. S7. Proline uptake by 22Δ8AA and 22Δ10α expressing UMAMIT14 and/or AAP3.

Fig. S8. Amino acid content in *umamit* mutants.

Fig. S9. Phloem transfer assay.

Fig. S10. *umamit14-1* root growth in different nitrogen regimes.

Fig. S11. Xylem amino acid content in the wild-type and the *umamit* mutants.

Table S1. List of primers used.

Table S2. Relative abundance of amino acids secreted by yeast expressing UMAMIT14 and UMAMIT18.

Table S3. Physiological traits of *umamit* mutants.

Table S4. Shoot and root amino acid content of *umamit* mutants.

Table S5. Amino acids secreted by *umamit* mutants.

Table S6. CPM and dry weight of samples used in the shoot-to-root transfer assay using [^3^H]Gln.

Table S7. CPM and dry weight of samples used in the shoot-to-root transfer assay using [^14^C]sucrose.

Supplementary Data
