# Supplementary material for: UMAMIT14 is an amino acid exporter involved in phloem unloading in Arabidopsis roots
Source: J Exp Bot. 2016 Nov 17;67(22):6385–97. doi: 10.1093/jxb/erw412 (PMC5181585; doi:10.1093/jxb/erw412)
Supplement: Supplementary Data [file supp_67_22_6385__index.html]

UMAMIT14 is an amino acid exporter involved in phloem unloading in Arabidopsis roots — Supplementary Data 

# UMAMIT14 is an amino acid exporter involved in phloem unloading in Arabidopsis roots

## Supplementary Data

Data files

- Supplementary\_figures\_S1\_S11.pdf - Supplementary Data
- Supplementary\_tables\_S1\_7.pdf - Supplementary Data
