# Supplementary material for: UMAMIT14 is an amino acid exporter involved in phloem unloading in Arabidopsis roots
Source: J Exp Bot. 2016 Nov 17;67(22):6385–97. doi: 10.1093/jxb/erw412 (PMC5181585; doi:10.1093/jxb/erw412)
Supplement: Supplementary Data [file supp_erw412_Supplementary_figures_S1_S11.pdf]

Usually Multiple Acids Move In and out Transporters 14 is an Arabidopsis amino acid exporter involved in phloem unloading of amino acids in roots

Supplemental figures S1-S11

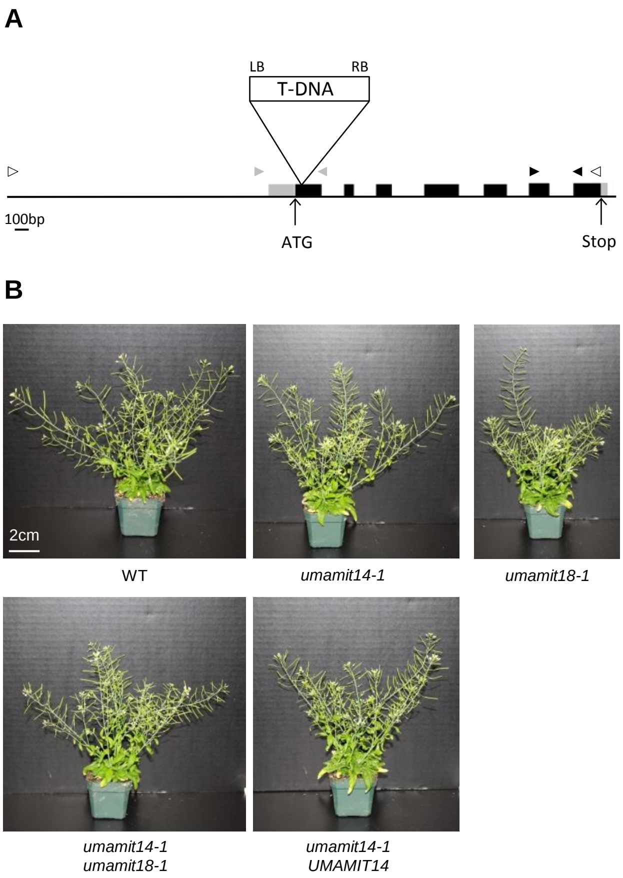

Figure S1. *umamit14-1* and *umamit18-1* T-DNA insertion mutants. (A) Location of the T-DNA insertion in *umamit14-1.* Grey and black boxes represent untranslated and translated regions of exons, respectively. Closed arrowheads represent the positions of forward and reverse primers used for qRT-PCR. Open arrowheads represent the positions of the primers used for creation of the complemented line. Grey arrowheads represent the position of the primers used for genotyping the T-DNA insertion. (B) Phenotypes of six-week-old wild type and *umamit* mutants grown in long day conditions.

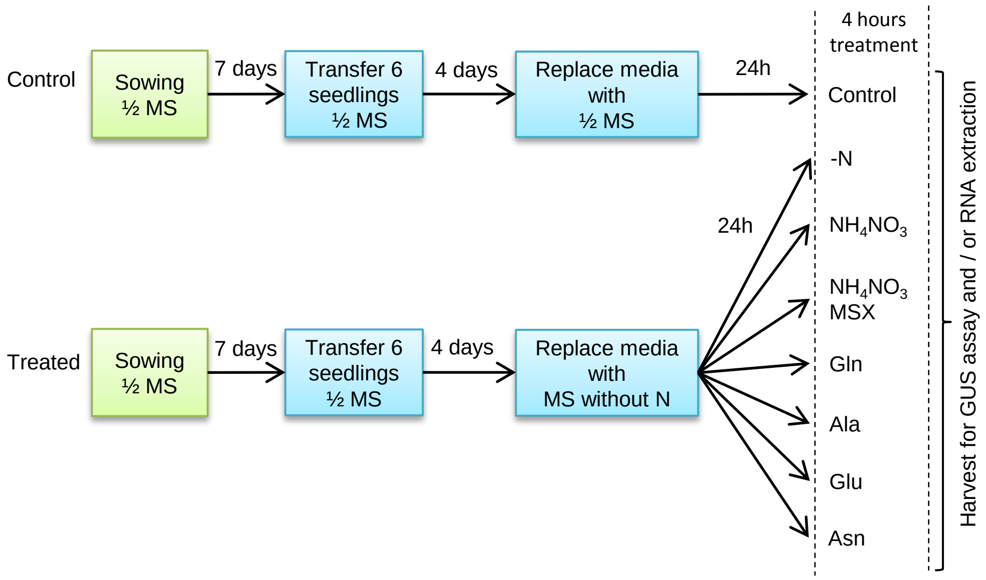

Figure S2: Nitrogen starvation and recovery experiment setup. These conditions were used to obtain the results shown on Figure 4. Green boxes and blue boxes represent solid and liquid media, respectively. ½ MS: Half strength Murashige and Skoog media. MS without N: full strength Murashige and Skoog media without nitrogen. Control treatment: ½ MS. MSX: methionine sulfoximine used at 1mM. For liquid growth conditions and treatments, plants were grown in 1 mL of liquid medium in a 24-well plate. All nitrogenous treatments were applied at 10 mM except Ala and Glu (20 mM), to match the nitrogen molarity of the other compounds used.

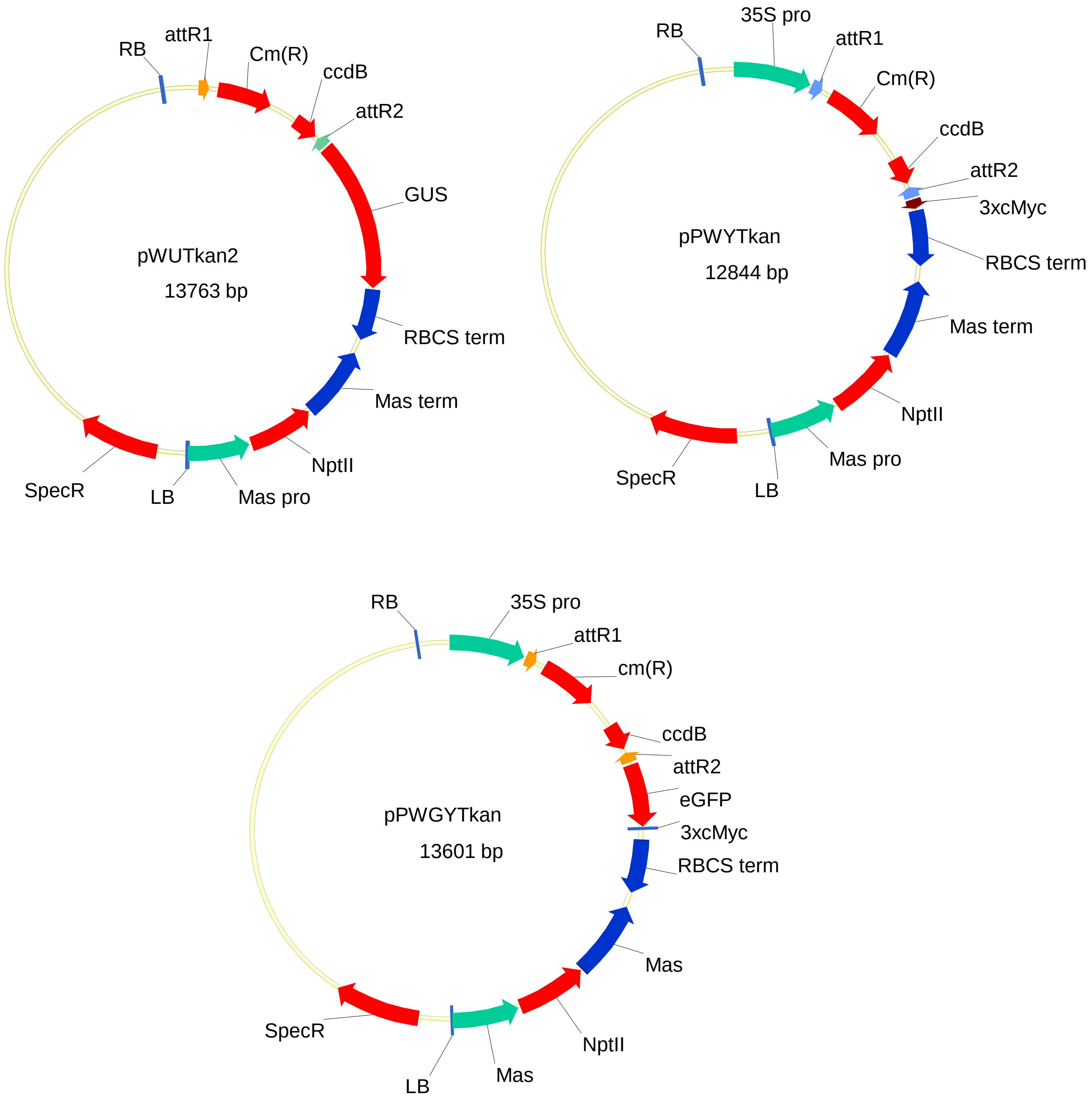

Figure S3. Maps of plant vectors used. Vectors pWUTkan2, pPWYTkan and pPWGYTkan used for creating promoter-GUS, UMAMIT14promoter-UMAMIT14cDNA-venus, and gene-GFP fusion, respectively. 35S pro: CaMV 35S promoter; attR1 and attR2: gateway recombination sites; ccdB: suicide gene; Cm(R): chloramphenicol resistance gene; cMyc: cMyc epitope; eGFP: enhanced GFP gene; GUS: uidA from *E. coli*; LB: T-DNA left border; Mas term: mannopine synthase terminator; Mas pro: mannopine synthase promoter; NptII: neomycin phosphotransferase; RB: T-DNA right border; RBCS term: Rubisco terminator from pea; SpecR: spectinomycin resistance gene.

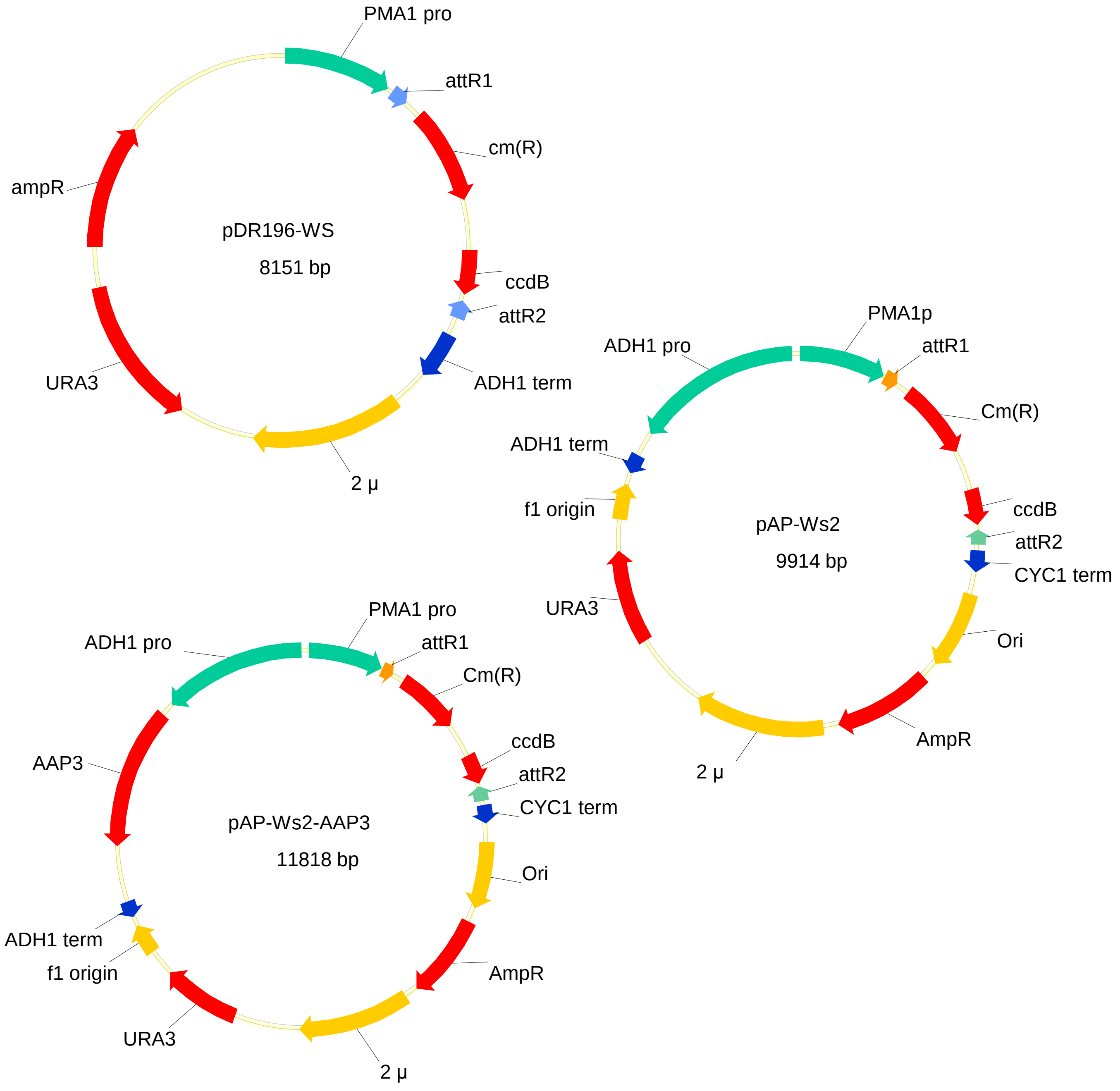

Figure S4. Maps of yeast vectors used. Vectors pDR196-Ws, pAPWs2-AAP3 and pAP-Ws. 2μ: 2μ replication origin for yeast; AAP3: Arabidopsis amino acid permease 3; ADH1 pro and term: alcohol dehydrogenase promoter and terminator; AmpR: ampicillin resistance gene; attR1 and attR2: gateway recombination sites; ccdB: suicide gene; Cm(R): chloramphenicol resistance gene; CYC term: terminator of the cytochrome C gene; f1 origin: replication origin for the f1 phage; Ori: replication origin in E. coli; PMA1 pro: plasma membrane ATPase promoter; URA3: uracil selection marker.

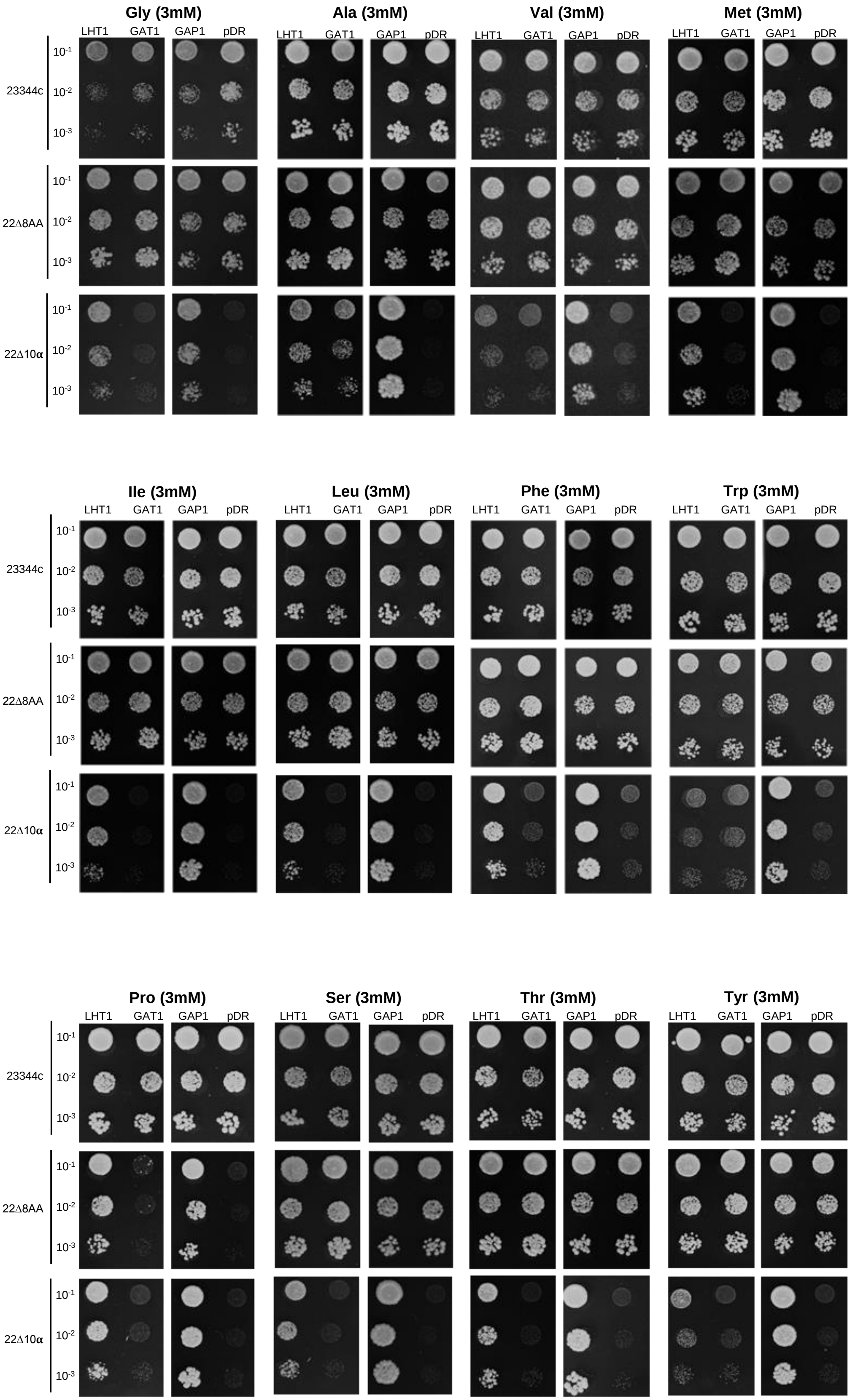
Gly (3mM)
Ala (3mM)
Val (3mM)
Met (3mM)
Ile (3mM)
Leu (3mM)
Phe (3mM)
Trp (3mM)
Pro (3mM)
Ser (3mM)
Thr (3mM)
Tyr (3mM)
LHT1
GAT1
GAP1
pDR
23344c
22Δ8AA
22Δ10α
10-1
10-2
10-3

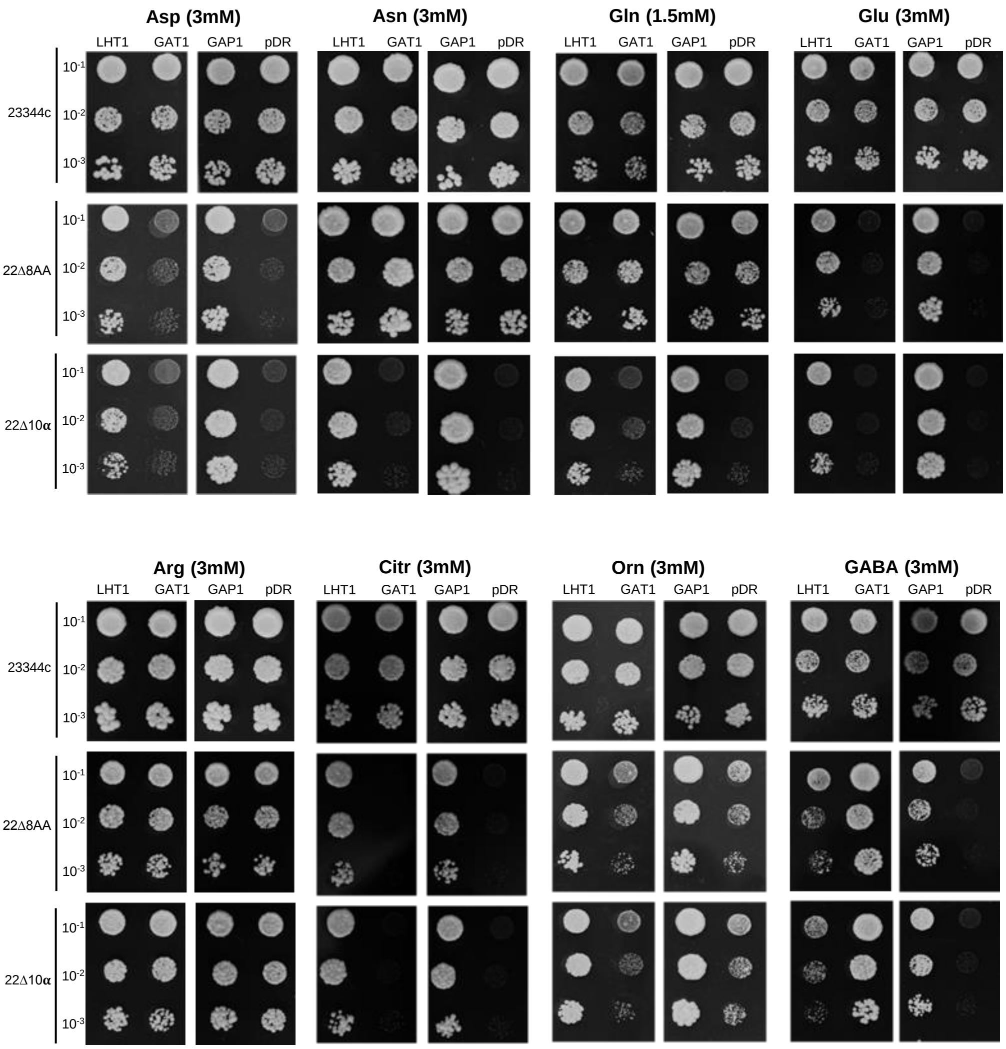

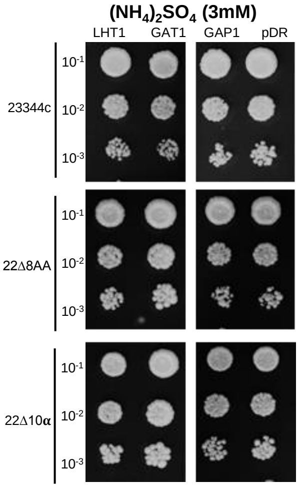

Figure S5. Yeast growth complementation assay. The media contains amino acids as the sole nitrogen source. The parental strain 23344c, 22Δ8AA and 22Δ10α were transformed with the vector pDR196-Ws containing the cDNAs of LHT1 (Lysine Histidine Transporter1; AT5G40780), GAT1 (GABA Transporter1; AT1G08230), GAP1 (General Amino acid Permease1; YKR039W) and the empty pDR196 vector. Yeast cells were grown overnight in selective medium, brought to OD=0.1, and diluted to OD=0.01 and 0.001. Drops of 4 µl were aligned on minimum media containing the mentioned concentration of amino acids. Pictures were taken after 5 days of growth at 30°C.

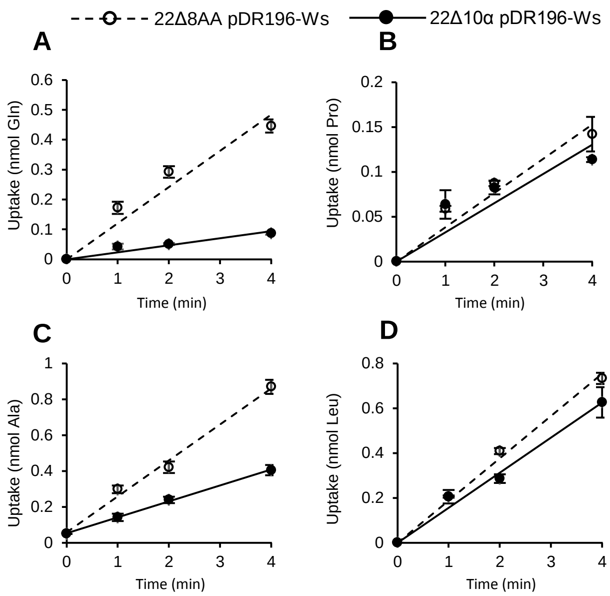

Figure S6. Amino acid uptake by 22Δ8AA and 22Δ10α. Uptake by yeast cells expressing pDR196-Ws empty vector from which the gateway cassette has been removed. Uptake was measured with 2 mM Gln (A), Pro (B), Ala (C) or Leu (D) . n=3 technical replicates.

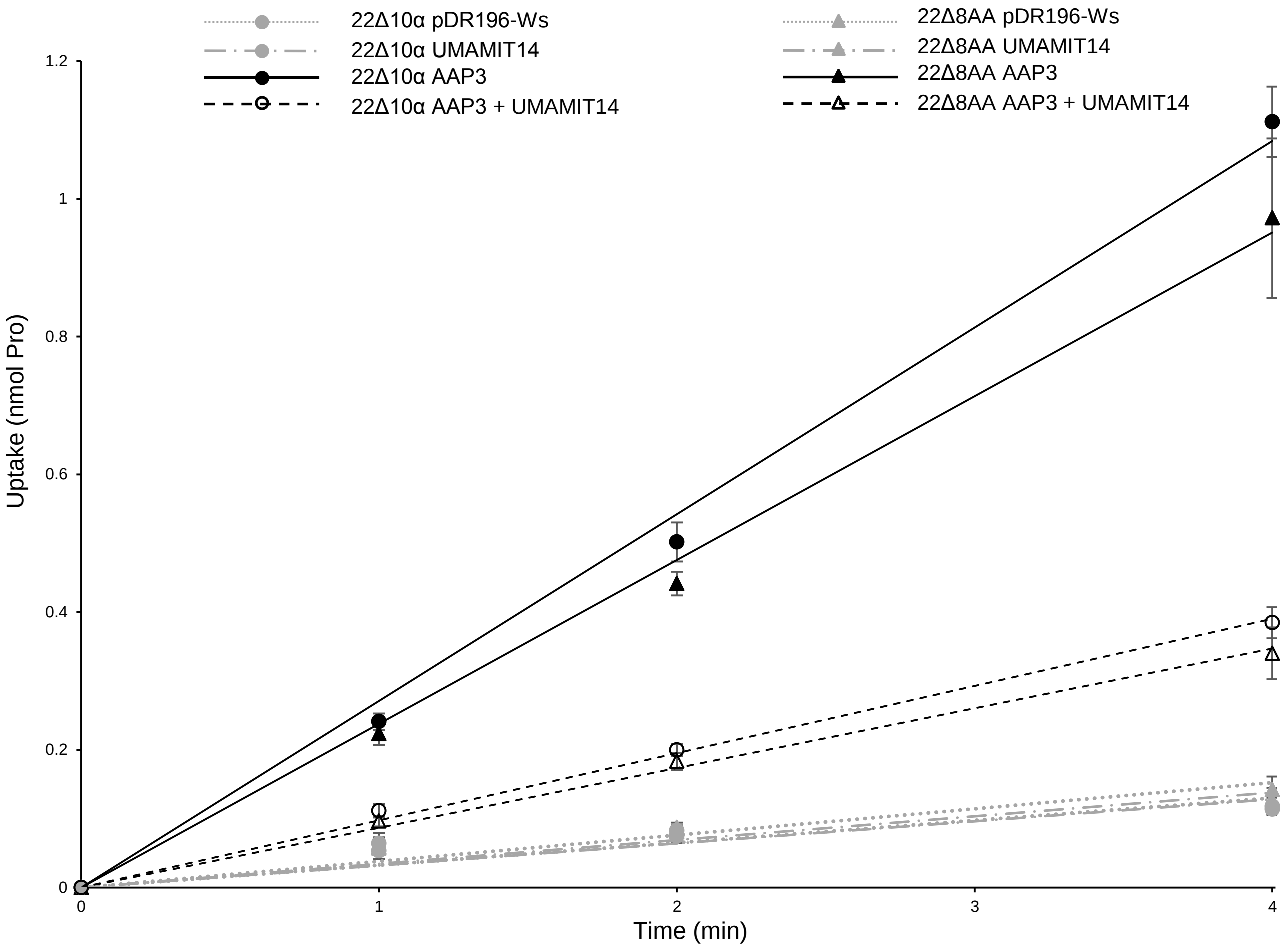

Figure S7. Proline uptake by 22Δ8AA and 22Δ10α expressing UMAMIT14 and/or AAP3. pDR196-Ws: cells transformed with empty pDR196-Ws, UMAMIT14: cells expressing UMAMIT14, AAP3: cells expressing AAP3, AAP3 + UMAMIT14: cells co-expressing AAP3 and UMAMIT14 carried on a single vector. Uptake was examined for 2 mM Pro (n=3 replicates).

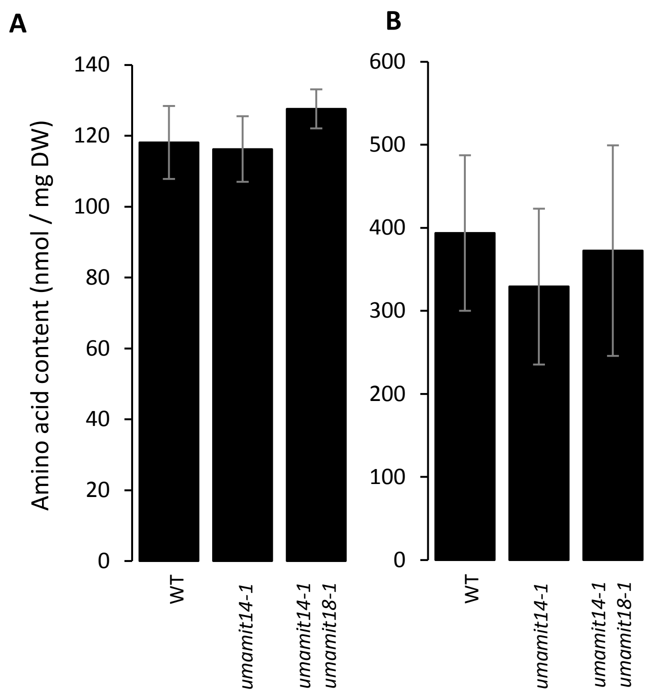

Figure S8. Amino acid content in *umamit* mutants. (A) shoots and (B) roots amino acid content of five week-old Arabidopsis plants grown in hydroponic conditions. Error bars correspond to standard deviation (n=3 biological replicates). No significant differences were found according to one-way ANOVA in conjunction with Tukey's test ($p<0.05$). Contents in individual amino acids from the same dataset are presented in Table S4.

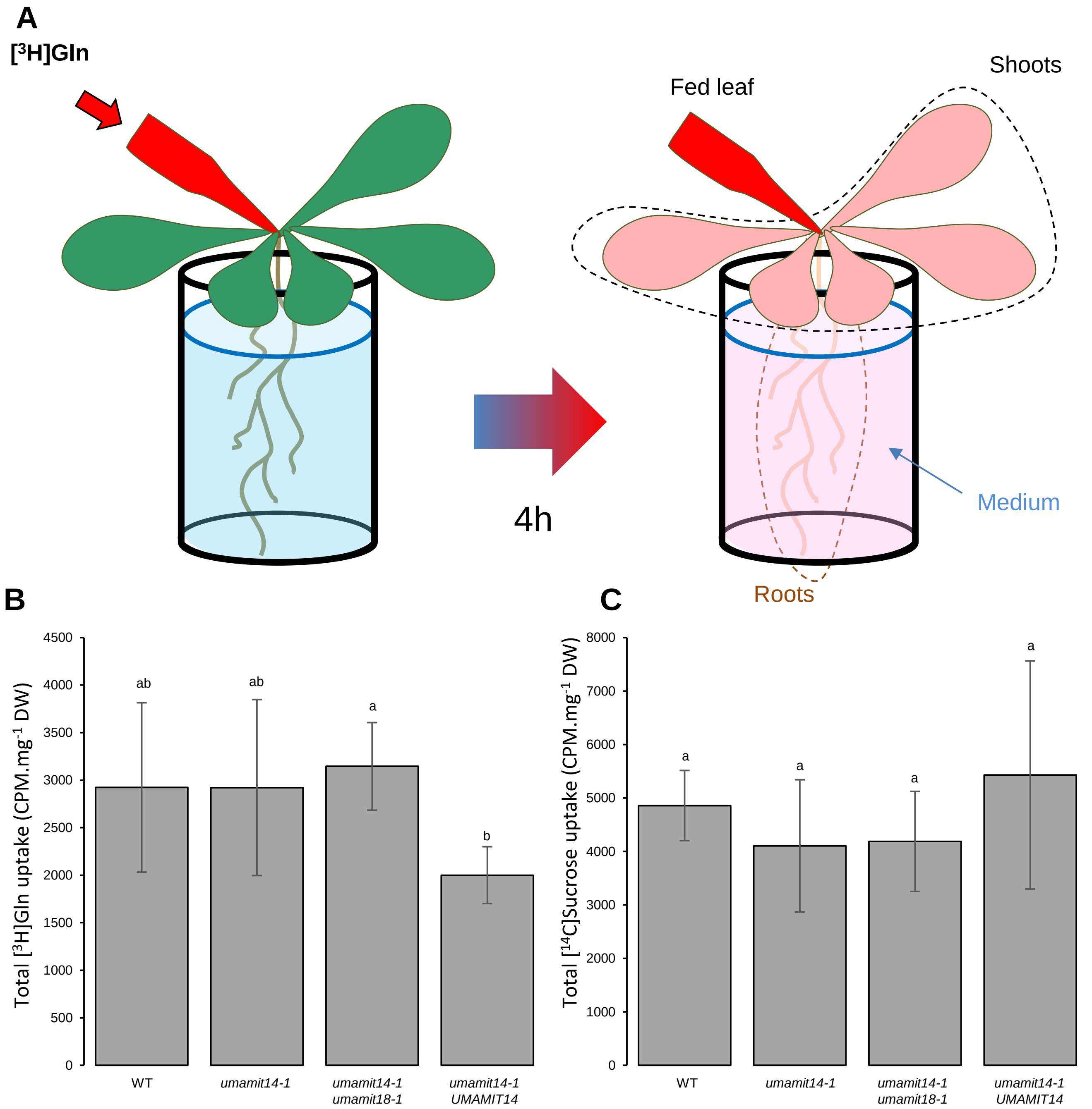

Figure S9. Phloem transfer assay. (A) Set up. The names of the different samples corresponds to the legend of Figure 6. (B) Glutamine and (C) Sucrose total uptake by plants. CPM: count per minute. Total uptake is the sum of CPM found in each sample for a given plant (Fed lead, shoots, roots and medium) divided by the plant dry weight. Significant differences ($p<0.05$) are indicated by different letters according to one way ANOVA in conjunction with Tukey's test (n=3 biological replicates). Raw data of the phloem translocation assay are presented in Table S5 and S6.

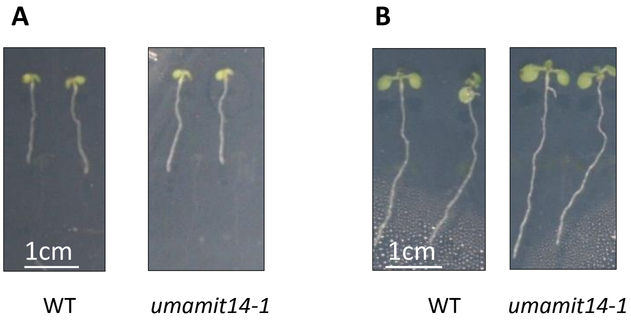

Figure S10. *umamit14-1* root growth in different nitrogen regimes. One-week-old Arabidopsis seedlings grown vertically on solid J medium without sugar, containing 0.1 (A) or 10 mM KNO_3 (B) .

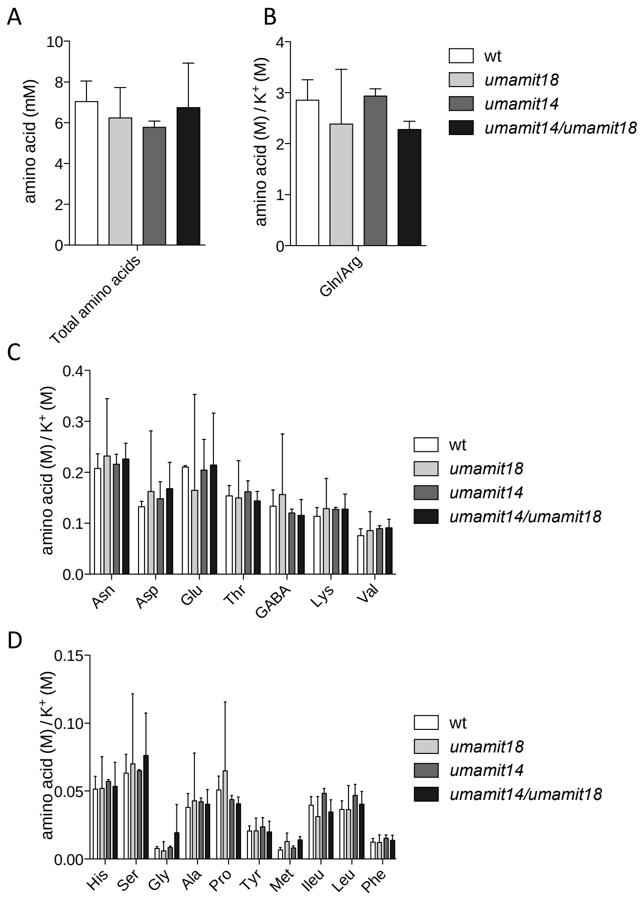

Figure S11. Xylem amino acid content in WT and *umamit* mutants. (A) Total amino acid concentrations. (B) to (D) Concentrations of individual amino acids, normalized by the K^+ concentrations. Gln/Arg (B); Asn, Asp, Glu, Thr, GABA, Lys, Val (C); and His, Ser, Gly, Ala, Pro, Tyr, Met, Ile, Leu, Phe (D). Xylem sap was extracted from decapitated 55-day-old plants grown in short day conditions. Error bars correspond to standard deviation (n=3 biological replicates). No significant difference was found according to one way ANOVA in conjunction with Tukey's test ($p<0.05$).
