# Supplementary material for: UMAMIT14 is an amino acid exporter involved in phloem unloading in Arabidopsis roots
Source: J Exp Bot. 2016 Nov 17;67(22):6385–97. doi: 10.1093/jxb/erw412 (PMC5181585; doi:10.1093/jxb/erw412)
Supplement: Supplementary Data [file supp_erw412_Supplementary_tables_S1_7.pdf]

Usually Multiple Acids Move In and out Transporters 14 is an Arabidopsis amino acid exporter involved in phloem unloading of amino acids in roots

Supplemental tables S1-7

Table S1. List of primers used. Primers sequences are given from 5’ to 3’. Underlined lower case represents bases added for the creation of *attB1* and *attB2* in forward and reverse primers, respectively.

Name	Type	Sequence
UMAMIT14 promoter	Forward	acaagtttgtacaaaaaagcaggcttcCACTAAAATATTTACATTGACC
	Reverse	accactttgtacaagaaagctgggtCTCATGTTTTTAAAGCCATATCTCAACGTAG
UMAMIT14 promoter (for modifying pPWYTkan)	Forward	GAGAAGATCTCTCGAGGTACAAAAAAGCAGGCTGGCACTAAAATATTTACATTGACC
	Reverse	TATAGAGCTCTGTTTTTAAAGCCATATCTCAACGTAG
UMAMIT14 genomic	Forward	acaagtttgtacaaaaaagcaggcttcGATATGGCTTTAAAAACATGGAAG
	Reverse	accactttgtacaagaaagctgggtCTCAGACTGATTCATTGGTGTTAGGCCT
UMAMIT14 CDS	Forward	gacaagtttgtacaaaaaagcaggcttcAGATATGGCTTTAAAAACATGGAAG
	Reverse	gaccactttgtacaagaaagctgggtACYAGACTGATTCATTGGTGTTAGGCCT
UMAMIT14 CDS (venus fusion)	Forward	GAGAGTCGACACCATGGCTTTAAAAACATGGAAG
	Reverse	ATATAGCGCTTGCGACTGATTCATTGGTGTTAGG
UMAMIT18 CDS	Forward	gacaagtttgtacaaaaaagcaggcttcAATAAAGATGAAAGGTGGAAGCATG
	Reverse	gaccactttgtacaagaaagctgggtACYAGGTACTGGTAACCACACCGTTAGT
UMAMIT14 T-DNA detection	Forward	CTATCAAAATTGCATTACTTAGTGG
UMAMIT14 T-DNA detection	Reverse	GGCGATACGACGCTAGAACGTGAG
Actin2 qPCR	Forward	GGTAACATTGTGCTCAGTGGTGG
	Reverse	AACGACCTTAATCTTCATGCTGC
UMAMIT14 qPCR	Forward	ATGGTCATTGTTGCGATCTT
	Reverse	TGGTTCGTCTTTGCTTTTTC

Table S2. Relative abundance of amino acids secreted by yeast expressing UMAMIT14 and UMAMIT18. Amino acid contents represented in Figure 2 were used. The average of content obtained for the empty plasmid was subtracted to the content of each biological replicates of UMAMIT14 and UMAMIT18. Each value, which represents amino acid secretion above the background level, was divided by the sum of these normalized values, and expressed as a percentage. Stars indicate significant difference between UMAMIT14 and UMAMIT18 results (t-test; p-value<0.5). GABA: gamma-amino-butyric acid.

	Gln+ Arg*	Ala*	Glu*	Ser*	Gly*	Asn	Asp*	Pro*	Thr	Val*	Cys	His	Lys	Ile*	Leu*	Met	GABA	Phe
UMAMIT18	77.8	5.8	3.7	1.8	1.8	1.7	-0.4	1.1	2.3	2.0	0.5	0.7	0.0	0.5	0.2	0.0	0.0	0.06
UMAMIT14	43.3	27.6	7.7	5.9	4.6	2.0	1.1	2.5	2.5	1.7	0.0	0.4	0.0	0.4	0.3	0.0	0.0	0.06

Table S3. Physiological traits of *umamit* mutants. Characteristics of nine-week-old Arabidopsis plants grown in soil in long day conditions. Biomass represents all plant tissue collected from the aerial parts, minus the seeds. Significant differences ($p<0.05$) are indicated by different letters according to one-way ANOVA in conjunction with Tukey's test (n=4 biological replicates).

	Plant size (cm)	Biomass (mg)	Weight of 100 seeds (mg)	Number of seeds (thousands)	Number of seeds per silique	%C in seeds	%N in seeds	Seed protein content (µg.mg^{-1} DW)
WT	36.5 (a)	657 (a)	2.08 (a)	134 (a)	49.8 (a)	55.9 (a)	4.82 (a)	134.5 (a)
umamit14-1	35.5 (a)	400 (ab)	2.31 (a)	165 (a)	39.7 (a)	55.2 (a)	4.72 (a)	152.5 (a)
umamit18-1	28.2 (ab)	400 (ab)	1.97 (a)	118 (a)	44.5 (a)	54.4 (a)	4.58 (a)	170.6 (a)
umamit14-1 umamit18-1	27.6 (ab)	396 (ab)	2.15 (a)	194 (a)	41.4 (a)	54.9 (a)	4.50 (a)	124.3 (a)
umamit14-1 UMAMIT14	32.5 (a)	800 (a)	2.26 (a)	111 (a)	45.4 (a)	55.3 (a)	4.98 (a)	137.2 (a)

Table S4. Shoot and root amino acid content of *umamit* mutants. Amino acids were extracted from five-week-old Arabidopsis plants growing in hydroponic conditions. Values are expressed in nmol.mg^{-1} DW. Significant differences ($p<0.05$) are indicated by different letters according to ANOVA in conjunction with Tukey's test (n=4 biological replicates). n.d.: not detected.

	Genotype	Ala	Asn	Asp	GABA	Gln	Glu	Gly	His	Ile	Leu	Phe	Pro	Ser	Thr	Val	Total
Shoots	WT	1.24 (a)	6.20 (a)	9.29 (a)	0.78 (a)	22.6 (a)	10.6 (a)	18.0 (a)	1.32 (a)	1.88 (a)	2.56 (a)	1.96 (a)	2.55 (a)	30.5 (a)	6.16 (a)	2.61 (a)	118.1 (a)
	umamit14-1	1.53 (a)	6.32 (a)	11.1 (a)	0.46 (b)	22.2 (a)	12.2 (ab)	15.0 (a)	1.24 (a)	1.79 (a)	2.33 (a)	1.77 (a)	2.02 (a)	29.8 (a)	6.07 (a)	2.52 (a)	116.2 (a)
	umamit14-1 umamit18-1	2.81 (b)	7.29 (a)	12.8 (b)	0.61 (a)	23.3 (a)	14.8 (b)	15.5 (a)	1.22 (a)	1.63 (a)	2.35 (a)	1.85 (a)	1.89 (a)	33.4 (a)	5.53 (b)	2.53 (a)	127.6 (a)
Roots	WT	121 (a)	48.2 (a)	19.3 (a)	28.2 (a)	47.1 (a)	62.1 (a)	4.92 (a)	n.d.	2.40 (a)	4.39 (a)	0.92 (a)	9.12 (a)	27.2 (a)	13.1 (a)	5.45 (a)	393.8 (a)
	umamit14-1	38.5 (b)	50.4 (a)	25.1 (ab)	19.5 (a)	65.3 (a)	53.3 (a)	4.31 (a)	n.d.	3.02 (ab)	6.43 (a)	1.53 (a)	10.9 (a)	33.3 (a)	11.0 (a)	6.87 (a)	329.3 (a)
	umamit14-1 umamit18-1	59.8 (ab)	60.1 (a)	29.4 (b)	18.1 (a)	71.7 (a)	56.2 (a)	4.15 (a)	n.d.	4.71 (b)	5.79 (a)	1.25 (a)	13.1 (a)	29.9 (a)	11.5 (a)	6.91 (a)	372.6 (a)

Table S5. Amino acid secreted by *umamit* mutants. The data are the ones used in Figure 6. Plants were grown for two weeks in liquid J medium supplemented with 20 mM NH_4NO_3; the medium was replaced with fresh J medium and collected after three days for analysis. Values are expressed in nmol.mg^{-1} DW. Significant differences ($p<0.05$) are indicated by different letters according to one-way ANOVA in conjunction with Tukey's test (n=6 biological replicates).

	Ala	Asn	Asp	Gaba	Gln	Glu	Gly	His	Ile	Leu	Phe	Ser	Thr	Val	Total
Wild Type	1.26 (a)	7.74 (a)	1.03 (a)	1.64 (a)	15.3 (a)	3.16 (a)	3.23 (a)	0.28 (a)	0.12 (a)	0.14 (a)	0.15 (a)	0.80 (a)	0.46 (a)	0.50 (a)	35.43 (a)
umamit14-1	1.09 (a)	5.83 (b)	0.86 (a)	0.98 (b)	10.4 (b)	2.57 (b)	2.45 (b)	0.21 (b)	0.08 (b)	0.09 (b)	0.10 (a)	0.56 (b)	0.32 (b)	0.11 (a)	26.80 (b)
umamit18-1	0.79 (b)	6.49 (a)	0.85 (a)	1.11 (b)	8.11 (b)	2.48 (b)	2.67 (b)	0.29 (a)	0.07 (b)	0.08 (b)	0.16 (a)	0.57 (b)	0.30 (b)	0.10 (b)	24.04 (b)
umamit14-1 umamit18-1	0.34 (c)	3.07 (d)	0.71 (b)	0.47 (b)	1.90 (c)	1.74 (c)	2.32 (b)	0.29 (a)	0.02 (c)	0.02 (c)	0.13 (a)	0.32 (c)	0.12 (c)	0.03 (c)	11.47 (c)

Table S6. CPM and dry weight of samples used in the shoot-to-root transfer assay using [^{3}H]Gln.

	Sample	CPM					Dry weight (mg)			
		Fed leaf	Shoot	Root	Medium	Total	Fed leaf	Shoot	Root	Total
WT	1	6908	716	24	88	7648	0.6	2.71	0.58	3.89
	2	11967	2680	259	185	14907	0.4	2.88	0.72	4
	3	11000	1702	88	156	12791	0.35	3.36	0.44	4.15
umamit14-1	1	9648	1884	81	36	11613	0.81	2.75	0.89	4.45
	2	8101	1588	61	41	9750	0.47	2.88	1.1	4.45
	3	13739	2803	138	51	16681	0.31	2.45	1.45	4.21
umamit14-1 umamit18-1	1	9374	3378	54	41	12807	0.71	2.72	1.19	4.62
	2	6877	3620	114	41.8	10611	0.29	1.9	1.34	3.53
	3	11742	3726	126	40.8	15595	0.33	2.97	0.96	4.26
	4	7623	2361	43	31	10027	0.34	2.24	0.97	3.55
umamit14-1 UMAMIT14	1	5729	1480	174	126	7383	0.62	2.44	1.01	4.07
	2	7836	1098	144	112	9078	0.34	3.45	1.14	4.93
	3	9343	953	77	89	10373	0.77	2.81	0.84	4.42

Table S7. CPM and dry weight of samples used in the shoot-to-root transfer assay using [^{14}C]sucrose. n.d. means the radioactivity found in the sample was similar to the background.

Sample		CPM					Dry weight (mg)			
		Fed leaf	Shoot	Roots	Medium	Total	Fed leaf	Shoots	Roots	Total
WT	1	5618	8982	3355	n.d.	17955	0.35	2.85	1.07	4.27
	2	8205	11955	1958	n.d.	22118	0.42	3.16	0.98	4.56
	3	8496	12928	2915	n.d.	24339	0.27	3.52	0.62	4.41
umamit14-1	1	6806	4677	1574	n.d.	13057	0.37	3.4	0.7	4.47
	2	5552	8440	2281	n.d.	16273	0.51	2.72	0.84	4.07
	3	11804	6834	1626	n.d.	20264	0.44	2.67	0.65	3.76
umamit14-1 umamit18-1	1	6707	9296	1625	n.d.	17628	0.48	2.56	0.83	3.87
	2	4679	4364	1805	n.d.	10848	0.41	2.27	0.79	3.47
	3	5687	9341	1926	n.d.	16954	0.36	2.21	0.9	3.47
umamit14-1 UMAMIT14-1	1	5743	8070	2193	n.d.	16006	0.43	2.97	0.85	4.25
	2	4589	10473	2482	n.d.	17544	0.37	2.7	0.67	3.74
	3	9563	11386	3097	n.d.	24046	0.33	2.33	0.41	3.07
